# Liver cirrhosis contributes to the disorder of gut microbiota in patients with hepatocellular carcinoma

**DOI:** 10.1002/cam4.3045

**Published:** 2020-04-12

**Authors:** Ruipeng Zheng, Guoqiang Wang, Zhiqiang Pang, Nan Ran, Yinuo Gu, Xuewa Guan, Yuze Yuan, Xu Zuo, He Pan, Jingtong Zheng, Fang Wang

**Affiliations:** ^1^ Department of Pathogen Biology College of Basic Medical Sciences Jilin University Changchun China; ^2^ Department of Interventional Therapy The First Hospital of Jilin University Changchun, Jilin China

**Keywords:** gut microbiota, hepatitis, hepatocellular carcinoma, liver cirrhosis

## Abstract

**Background:**

Gut microbiota (GM) of patients with liver cancer is disordered, and syet no study reported the GM distribution of liver cirrhosis‐induced HCC (LC‐HCC) and nonliver cirrhosis‐induced HCC (NLC‐HCC). In this study, we aimed to characterize gut dysbiosis of LC‐HCC and NLC‐HCC to elucidate the role of GM in the pathogenesis of HCC.

**Methods:**

A consecutive series of fecal samples of patients with hepatitis (24 patients), liver cirrhosis (24 patients), HCC (75 patients: 35 infected by HBV, 25 infected by HCV, and 15 with alcoholic liver disease), and healthy controls (20 patients) were obtained and sequenced on the Illumina Hiseq platform. The HCC group contains 52 LC‐HCC and 23 NLC‐HCC. Bioinformatic analysis of the intestinal microbiota was performed with QIIME and MicrobiomeAnalyst.

**Results:**

Alpha‐diversity analysis showed that fecal microbial diversity was significantly decreased in the LC group, and there were significant differences in 3 phyla and 27 genera in the LC group vs the other groups (the healthy, hepatitis, and HCC groups). Beta‐diversity analysis showed that there were large differences between LC and the others. Gut microbial diversity was significantly increased from LC to HCC. Characterizing the fecal microbiota of LC‐HCC and NLC‐HCC, we found that microbial diversity was increased from LC to LC‐HCC rather than NLC‐HCC. Thirteen genera were discovered to be associated with the tumor size of HCC. Three biomarkers (*Enterococcus*, *Limnobacter*, and *Phyllobacterium*) could be used for precision diagnosis. We also found that HBV infection, HCV infection, or ALD (alcoholic liver disease) was not associated with intestinal microbial dysbiosis in HCC.

**Conclusion:**

Our results suggest that GM disorders are more common in patients with LC‐HCC. The butyrate‐producing genera were decreased, while genera producing‐lipopolysaccharide (LPS) were increased in LC‐HCC patients. Further studies of GM disorders may achieve early diagnosis and new therapeutic approaches for HCC patients.

## Graphical abstract

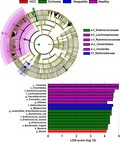

## 1 BACKGROUND

Liver cancer is the sixth malignancy and the fourth leading cause of cancer‐related death all over the word, with an estimated 841 000 new cases and 782 000 deaths annually.^1^ Hepatocellular carcinoma (HCC) is the most commonly diagnosed form of liver cancer, accounting for 80% of primary liver cancer.^2^ More seriously, it is estimated that the new HCC cases were as high as 466 000 in China in 2015,^3^ which caused serious public health problems due to the high prevalence, high mortality rate, and poor prognosis.^4^ The 5‐year survival rate of patients with HCC is particularly poor, mainly because the lack of early diagnostic markers leads to most patients being in the advanced stage when diagnosed.^5^, ^6^ Therefore, it is urgent to identify novel early diagnostic markers of HCC to improve prognosis.

HCC is a typical result of chronic liver inflammation, driven by the vicious cycle of liver damage, inflammation and repair, and generally undergoes the progression of hepatitis, liver fibrosis, and liver cirrhosis (LC) to liver cancer.^2^, ^7^ The main pathogenic factors of HCC include hepatitis B virus (HBV) infection, hepatitis C virus (HCV) infection, alcoholism, nonalcoholic fatty liver disease (NAFLD), aflatoxin B1 inhalation, and other factors such as obesity.^8^, ^9^, ^10^ Indeed, approximately 70%‐90% of patients with HCC have the accompanying appearance of LC, which represents the final stage of liver injury and inflammation.^11^, ^12^ More importantly, it was reported that approximately one‐third of patients with compensated LC could develop HCC during their lifetime.^13^ Recent studies have found that chronic inflammation, oxidative stress, telomere shortening, and insulin resistance are the main mechanisms leading to the development of LC to HCC.^14^ Moreover, increased intestinal permeability, as the hallmark of LC, can lead to bacteria and their products [such as lipopolysaccharide (LPS)] in the intestine translocating into the liver, thereby promoting hepatocarcinogenesis through aggravating inflammation, liver injury, and fibrosis.^15^, ^16^ Dysregulated bile acids caused by gut bacteria have also been found to play important roles in the progression of LC to liver cancer.^17^ However, the exact mechanism by which LC develops into liver cancer remains to be further clarified.

Increasing clinical evidence has suggested that the human GM plays pivotal roles in maintaining the body's microecosystem balance.^18^, ^19^ Gut microbial dysbiosis has been demonstrated to be the crucial determinant and player in liver diseases, such as NAFLD,^20^ LC,^21^ and liver cancer.^4^ The liver is connected to the gut through the portal vein to transport biologically active substances such as bile acids, and the gut microbes and their metabolites are retrograde transported to the liver, activating the inflammatory reaction by activating toll‐like receptors (TLRs), aggravating liver damage, and accelerating disease progression.^19^ The qualitative and quantitative changes in gut microbiota composition (also known as a dysbiosis) have proven beneficial the development of NAFLD and its development to nonalcoholic steatohepatitis and HCC.^22^ Moreover, studies in animal models have also confirmed that GM could promote the deterioration of HCC through the gut‐liver axis, and the treatment of probiotics could regulate intestinal flora, affect intestinal immune status and inhibit tumor growth.^23^ However, recent research has found that host health does not depend on one or a few dominant organisms, but on the balance of the composition of the entire microbial community.^19^ This means that we must determine the GM composition of patients, which is fundamental to the treatment of HCC. Unfortunately, current research has only focused on the link between the GM and a specific liver disease and the associations among GM, the different types of liver diseases and the different causes have not yet been reported. Therefore, the role of GM in human hepatocarcinogenesis requires further investigation.

This study explored the GM of patients with hepatitis, LC, HCC (LC‐HCC and NLC‐HCC), and healthy controls with high‐throughput sequencing techniques to systematically differentiate the influence of different etiologies and different types of liver diseases on the intestinal microbiota. This would be helpful in finding early diagnostic markers of HCC disease and providing new insights for the treatment of liver diseases, especially HCC.

## 2 METHODS

### 2.1 Participant characteristics

Patients with hepatitis, LC, or HCC caused by infection with HBV, HCV, or ALD were prospectively recruited from March 2017 to April 2018 at the First Hospital of Jilin University. Fecal samples from the patients and age‐ and sex‐ matching healthy subjects were obtained. This study was performed in accordance with the Helsinki Declaration and Rules of Good Clinical Practice. It was approved by the First Hospital of Jilin University Ethics Committee (2017‐342) and registered in Clinical Registry Platform (Registry ID: ChiCTR‐ROC‐16010189). All participants signed written informed consent on enrolment.

Hepatitis, LC, and HCC were diagnosed using Magnetic Resonance (MR), Computed Tomography (CT), HE staining of pathological sections, serum AFP levels, and chronic liver disease history. Viral serologic testing including HBsAg and HCVAb was performed in all groups. Patients with HCC were classified as HBV‐HCC or HCV‐HCC based on viral serologic testing, and they were divided into LC‐HCC and NLC‐HCC according to the presence or absence of cirrhosis (as shown in Figures [Link], [Link], [Link], [Link], [Link], [Link], [Link], [Link] in supplementary materials). ALD was diagnosed according to the Guidelines of Prevention and Treatment for Alcoholic Liver Disease of China.^24^ Briefly, the history of alcohol consumption was more than 5 years and ethanol consumption was ≥40 g/d for men and ≥20 g/d for women. Healthy people were selected from people who came to our hospital for annual physical examinations, and all of the results, including serological tests, liver function, and computed tomography scan and other tests, were in the normal range.

The exclusion criteria were adopted in all groups and as shown in a previous study.^25^ Briefly, all participants had not received prior anticancer treatment; no other diseases, such as heart disease or hypertension, were present; and they did not take drugs such as antibiotics, prebiotics, or other drugs in the last 6 months. In addition, healthy participants who had intestinal and liver‐related diseases were also excluded.

### 2.2 Fecal sample collection and DNA extraction

Fresh tail stool samples of more than 5 g were obtained and immediately stored at −80°C in sterile containers until analysis. The total DNA was extracted with the CTAB DNA extraction method, and the concentration was detected. PCR was then performed in a PCR instrument (Applied Biosystems^®^ 2720) targeting the hypervariable V4 region of the 16S rRNA gene with the forward primer: 341F (5′‐CCTAYGGGRBGCASCAG‐3′) and the reverse primer: 806R (5′‐GGACTACNNGGGTATCTAAT‐3′). The PCR conditions were as follows: 94°C for 2 minutes; 30 cycles of 94°C for 30 seconds, 55°C for 45 seconds, and 72°C for 30 seconds; and completed with the final elongation step at 72°C for 5 minutes. PCR products were quantified using a Quant‐iT PicoGreen dsDNA kit (Invitrogen) and detected using a 2% (w/v) agarose gel. Then the strips were purified with AxyPrepDNA Gel (Axygen). A sequencing library was constructed according to the manufacturer's protocol, and sequencing was conducted on the Illumina Hiseq 2500 platform.

### 2.3 16S rRNA data analysis

The raw sequencing data for all samples were deposited into the NCBI Sequence Read Archive database (Accession number, PRJNA540574). The raw reads were quality filtered with QIIME2 (v2018.11.4). Noisy sequencing data, including error tags, chimera, and low‐quality sequences, were excluded. The clean data were clustered into operational taxonomic units (OTUs) at the 97% threshold. OTUs were performed against Greengenes Databases (Release 13.8) and the rare OTUs (≤0.001%) were filtered. The alpha and beta diversity, including principal coordinate analysis (PCoA) and nonmetric multidimensional scaling (NMSD), were calculated with QIIME and MicrobiomeAnalyst Platform according to the relative abundance of OTUs. The different taxonomies were also identified with the linear discriminant analysis (LDA) effect size (LEfSe). Moreover, Redundancy analysis (RDA) was performed according to the different critical clinical factors using the R vegan packages.

### 2.4 Statistical analysis

The results are expressed as the mean ± SD. The normal distribution was performed using the Kolmogorov‐Smirnov test and the homogeneity of the variance was analyzed with the *F *test. The statistical significance of the difference was estimated using unpaired two‐tailed Student's or Welch's *t* test in two‐group analyses. One‐way ANOVA was performed for analyses among multiple groups. Moreover, Fisher's exact test was used to compare the categorical variables. All statistical significance was accepted at *P* < .05. All analyses were performed with GraphPad Prism Software (Version 6; GraphPad) or R software (v3.6.1).

## 3 RESULTS

### 3.1 Demographics of the participants

**Figure 1:**
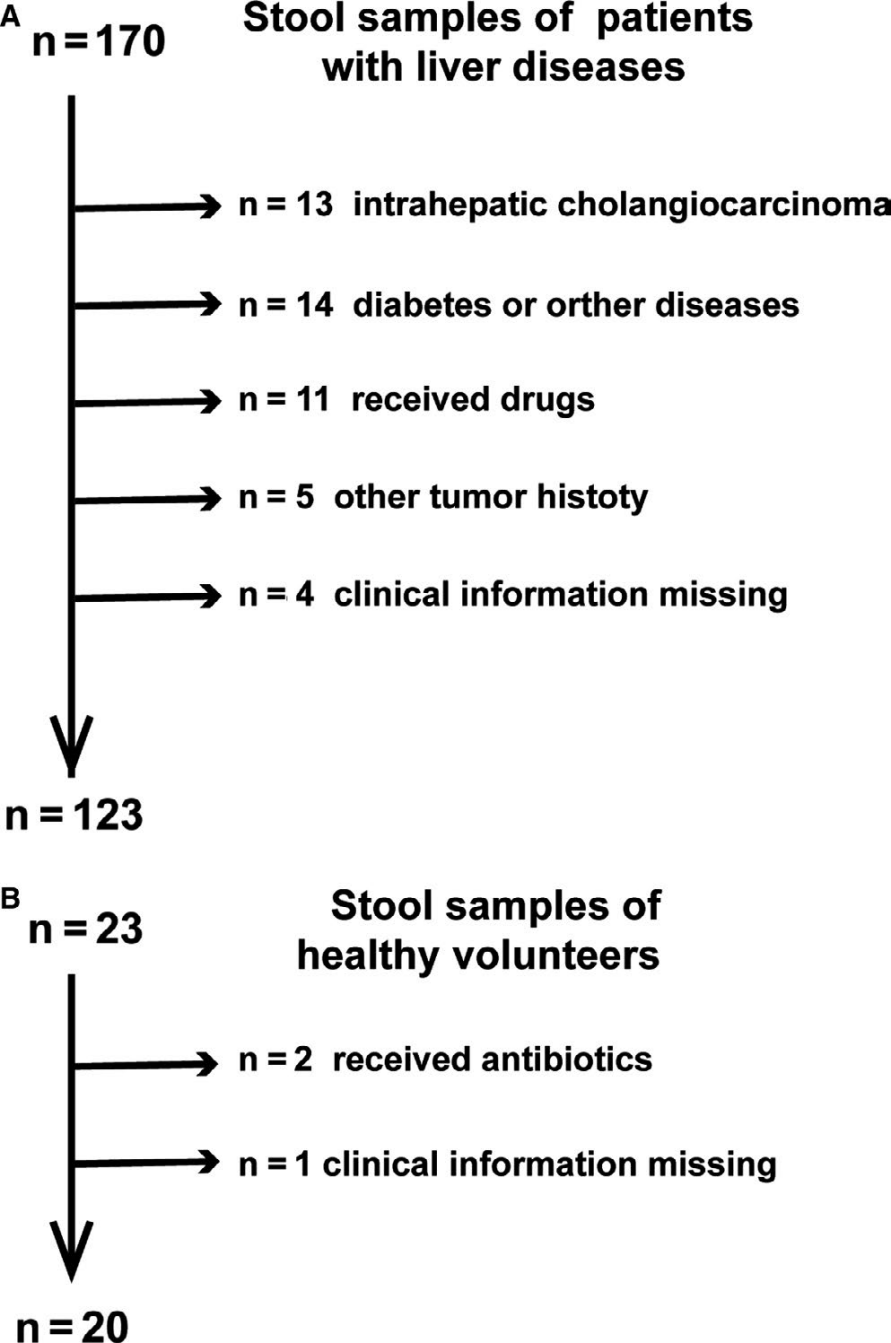
Study design and flow chart. A total of 143 fecal samples were collected from 123 patients and 20 healthy volunteers who were recruited for this study. A, Exclusion of patients with liver diseases. B, Exclusion of healthy volunteers

A total of 170 patients and 23 healthy volunteers were recruited in this study. After a strict screening process, 47 patients (13 with intrahepatic cholangiocarcinoma; 14 with heart disease, diabetes, or other diseases; 5 for proton pump inhibitors use; 6 for taking antibiotics; 5 with other tumor history; and 4 for missing clinical information) were included. Two healthy volunteers for taking antibiotics and one healthy volunteer for missing clinical information were excluded according to the exclusion criteria (Figure 1). Finally, 123 patients with liver diseases were divided into hepatitis (24 patients), LC (24 patients), and HCC (75 patients total; 35 HBV‐HCC, 25 HCV‐HCC, and 15 ALD‐HCC); 20 healthy people were recruited as the control group (Figure 1).

The clinical characteristics of the participants in this study are summarized in Table 1, and detailed clinical information of the participants is provided in Table S1. There were no significant differences among the four groups, including age, sex, and body mass index. Serum levels of alpha‐fetoprotein, alanine aminotransferase, aspartate aminotransferase, and glutamyl transpeptidase were markedly increased, while the concentrations of total protein and albumin were significantly decreased in patients with HCC vs the other groups (Table 1).

**Table 1:** Clinical characteristics of the enrolled participants

Clinical and pathological indexes	Healthy (n = 20)	Hepatitis (n = 24)	LC (n = 24)	HCC (n = 75)
Age (y)	56.70 ± 8.47	57.50 ± 7.62	58.08 ± 6.93	58.47 ± 8.78
Sex (female/male)	7/13	9/15	8/16	24/51
BMI	23.22 ± 1.94	22.58 ± 1.66	22.90 ± 1.59	22.53 ± 1.77
AFP (ng/mL)				
≦20	20 (100%)	21 (87.5%)	18 (75%)	19 (25.3%)
>20	0 (0%)	3 (12.5%)	6 (25%)	56 (74.7%)
Tumor size (cm)				
≦2	—	—	—	9 (12.0%)
2< &≦5	—	—	—	66 (88.0%)
Child‐Pugh				
A	—	24 (100%)	24 (100%)	60 (80.0%)
B	—	0 (0%)	0 (0%)	15 (20%)
ALT (9‐50 U/L)	24.0 ± 9.4	28.8 ± 9.3	34.2 ± 13.3	45.6 ± 44.3**
AST (15‐40 U/L)	23.5 ± 4.7	26.7 ± 7.8	33.9 ± 14.3	47.9 ± 38.2***
GGT (10‐60 U/L)	22.8 ± 5.2	31.7 ± 8.4	49.7 ± 15.6****	68.6 ± 61.9****
Total protein (65‐85 g/L)	72.9 ± 2.7	73.0 ± 3.0	72.0 ± 4.7	67.2 ± 5.9***
Albumin (40.0‐55.0 g/L)	49.3 ± 2.7	46.4 ± 7.5	43.8 ± 6.0.4	35.9 ± 5.8***
Globulin (20.0‐35.0 g/L)	26.0 ± 3.7	25.7 ± 3.5	29.7 ± 3.3**	29.2 ± 4.0*
Total bilirubin (6‐30 µmol/L)	16.1 ± 2.7	17.9 ± 4.2	21.2 ± 5.7*	18.4 ± 21.4*
Direct bilirubin (0‐8 µmol/L)	4.9 ± 1.2	5.4 ± 1.3	8.8 ± 2.4***	7.7 ± 6.4*
Dietary habit	Mixed diet	Mixed diet	Mixed diet	Mixed diet

Note

One‐way analysis of variance was used to evaluate the difference among the three groups. Categorical variables were compared with Fisher's exact test.

Abbreviations: AFP, alpha‐fetoprotein; ALT, alanine aminotransferase; AST, aspartate aminotransferase; BMI, body mass index; Child‐Pugh, Child‐Turcotte‐Pugh score; GGT, glutamyl transpeptidase; HCC, hepatocellular carcinoma; LC, liver cirrhosis.

Data are shown as the mean ± SD,

* *P*﹤0.05;

** *P*﹤0.01;

*** *P*﹤0.001.

**** *P*_<_0.0001.

### 3.2 Estimation of sequencing depth

**Figure 2:**
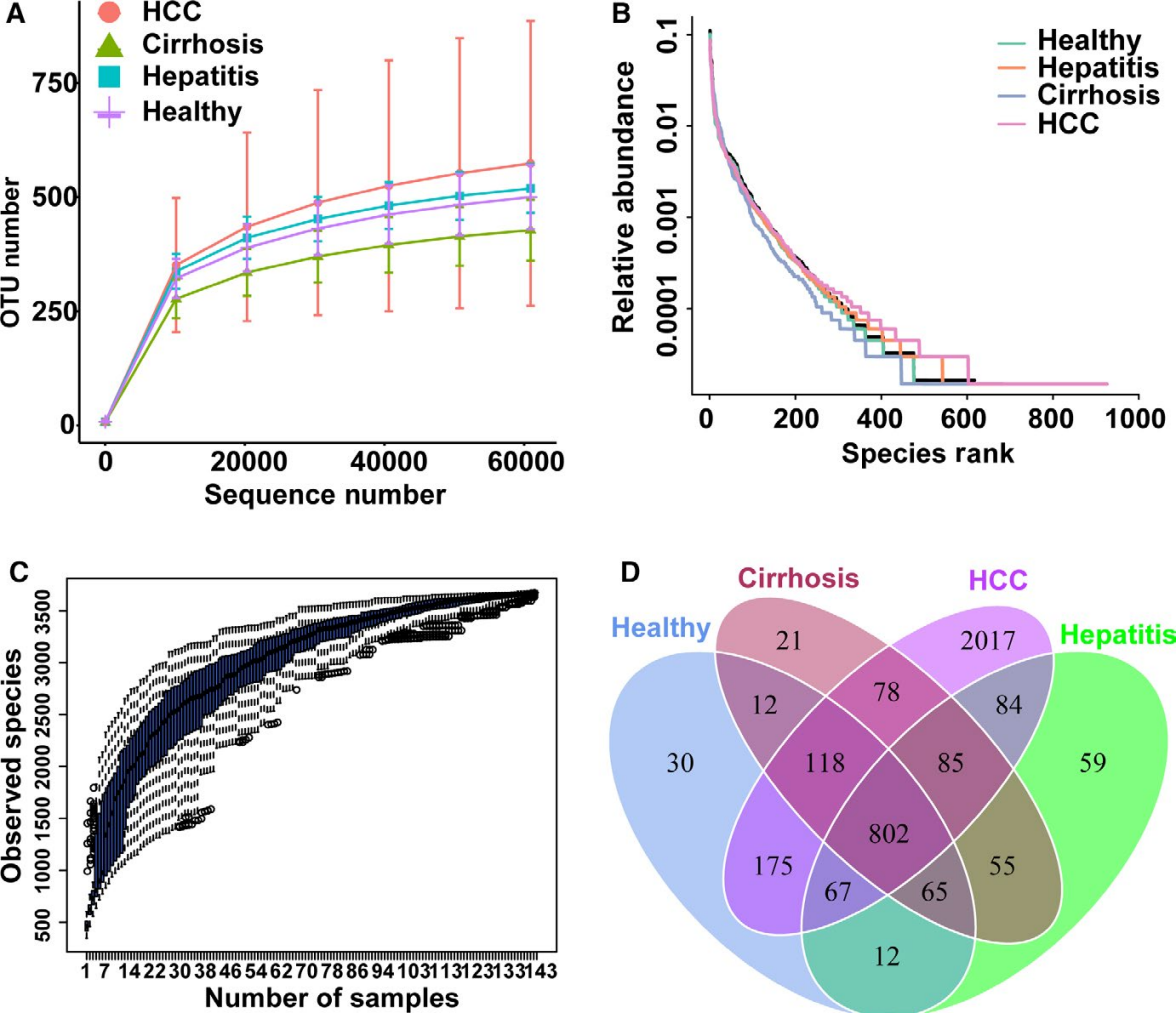
Estimation of sample depth and Venn diagram in the healthy, hepatitis, LC, and HCC groups. A, Rarefaction curves. B, Rank Abundance curves. C, Species Accumulation Boxplots of all groups. D, Venn diagram. LC, liver cirrhosis; HCC, hepatocellular carcinoma; OTUs, operational taxonomy units

Sequencing of 16S rDNA of 143 samples retrieved an overall number of 12 792 154 reads, 7 675 292 after filtering, which were clustered in 3724 OTUs. The rarefaction curves displaying the sequencing depth reached a plateau in each sample, indicating that the sequencing depth is sufficient (Figure 2A). The Rank Abundance curves reflected that the species in each group all have good richness and uniformity (Figure 2B). The Species Accumulation Boxplot showed that the estimates of cumulative genus richness reached asymptotic values, indicating that the sampling effort was great enough (Figure 2C). Furthermore, a Venn diagram displaying the overlaps among four groups showed that 802 of the total richness of 3504 OTUs were shared among the four groups, and 1083 of 3504 OTUs were shared between LC and HCC. However, 2017 of 3504 OTUs were unique to the HCC group, and only 21 of 3504 OTUs were unique to the LC group (Figure 2D).

### 3.3 Decreased gut microbial diversity in the LC group

**Figure 3:**
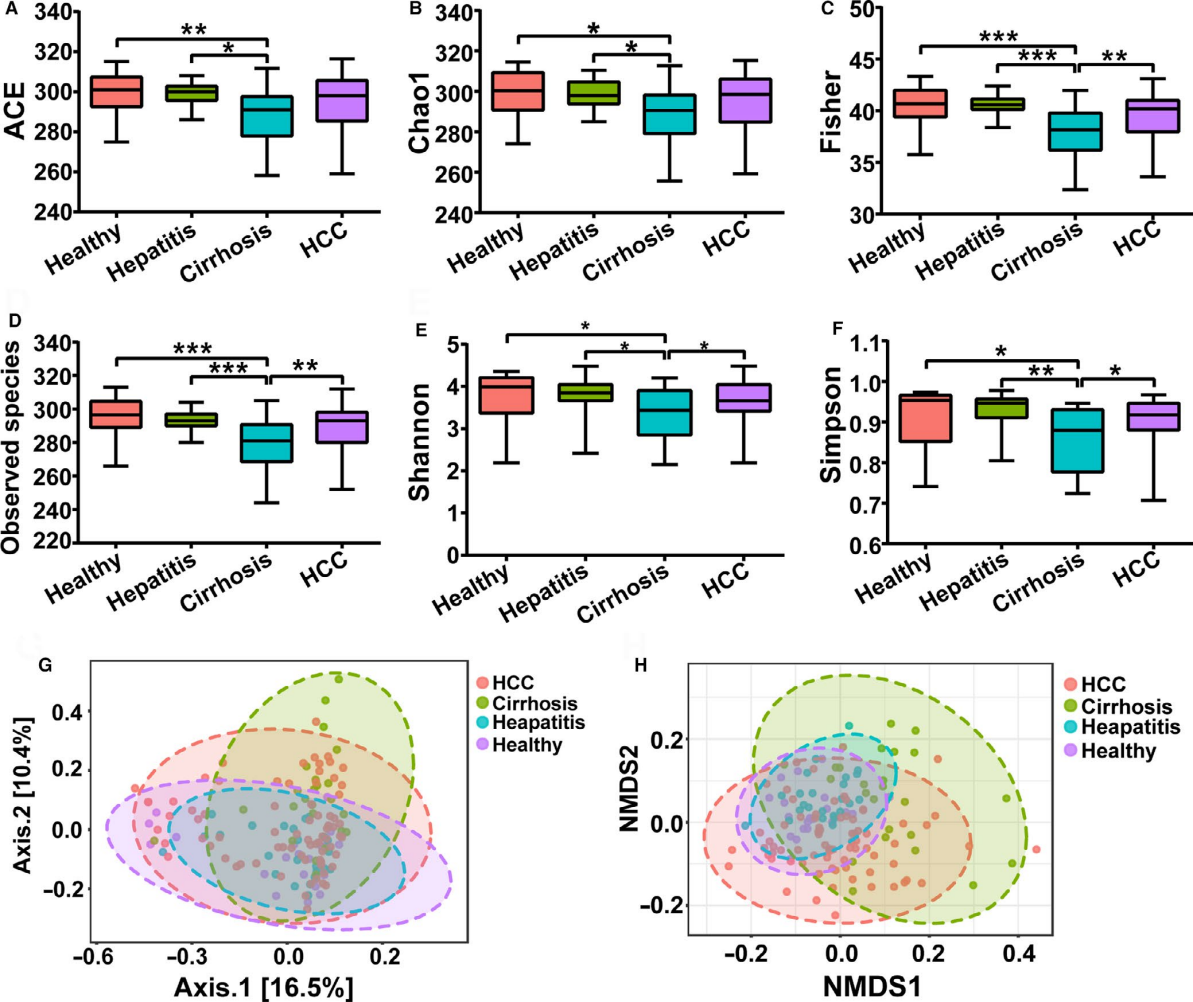
Alpha‐diversity analysis and beta‐diversity analysis among the healthy, hepatitis, LC, and HCC groups. A, ACE index. B, Chao1 index. C, Fisher index. D, Observed species index. E, Shannon index. F, Simpson index. G, PCoA analysis based on unweighted UniFrac distance matrix H, NMDS analysis. Data are shown as the mean ± SD, *P <* *.05; **P <* *.01; ***P <* *.001. LC, liver cirrhosis; HCC, hepatocellular carcinoma; PCoA, principal coordinates analysis; NMDS, nonmetric multidimensional scaling

Alpha‐diversity analysis showed a significant decrease in gut microbial diversity in the LC group, while no significant differences were found among the healthy, hepatitis and HCC groups (Figure 3). Compared with the healthy group, the richness of the GM showed a significant decrease in the LC group, which was estimated by the ACE index (*P *<* *.01), Chao1 index (*P *<* *.05), Fisher index (*P *<* *.001), and Observed species index (*P* < .001) (Figure 3A‐D). The evenness and richness of the GM were also evaluated by the Shannon index and the Simpson index, which showed a significant decrease in the LC group (*P* < .05) (Figure 3E,F). Interestingly, compared with the LC group, the gut microbial diversity of the HCC group was significantly increased according to the Fisher index (*P *<* *.01), Observed species index (*P *< .01), Shannon index (*P *< .05), and Simpson index (*P *<* *.05).

Moreover, beta‐diversity analysis was also performed through PCoA based on an unweighted UniFrac distance matrix and NMDS (Figure 3G,H). Similar to the alpha‐diversity analysis results, no significant clustering was observed among the healthy, hepatitis, and HCC groups. However, a significant clustering was formed as the LC group was distinguished from the other three groups to some extent. In summary, the results of the alpha‐diversity analysis and beta‐diversity analysis both showed that gut microbial diversity was decreased significantly in the LC group.

### 3.4 Phylum‐level and genus‐level changes in the GM in the four groups

**Figure 4:**
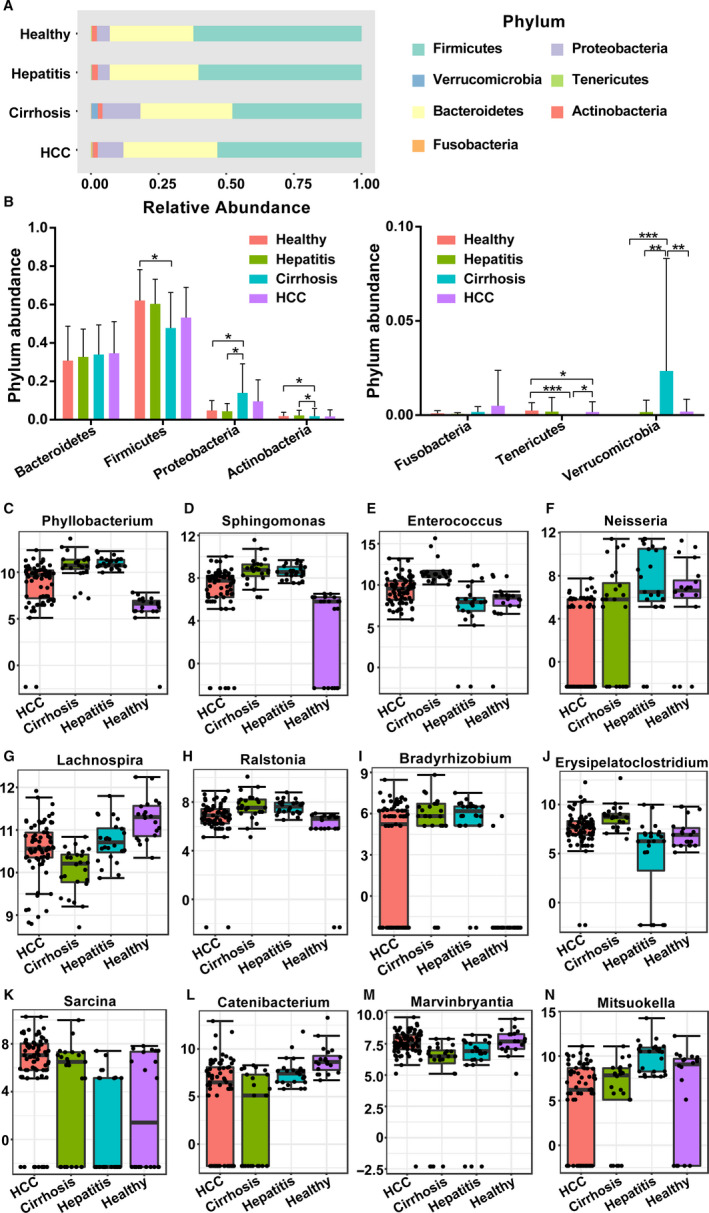
The differences in GM among the healthy, hepatitis, LC, and HCC groups at the phylum level and genus level. A,B, Composition of the GM at the phylum level. The top 12 taxonomies of GM at the genus level are shown as (C) *Phyllobacterium*, (D) *Sphingomonas*, (E) *Enterococcus*, (F) *Neisseria*, (G) *Lachnospira*, (H) *Ralstonia*, (I) *Bradyrhizobium*, (J) *Erysipelatoclostridium*, (K) *Sarcina*, (L) *Catenibacterium*, (M) *Marvinbryantia,* and (N) *Mitsuokella*. GM, gut microbiota; LC, liver cirrhosis; HCC, hepatocellular carcinoma

We next examined the composition differences in the GM at the phylum and genus levels in healthy, hepatitis, LC and HCC groups. A total of seven bacterial phyla, including Firmicutes, Proteobacteria, Verrucomicrobia, Tenericutes, Bacteroidetes, Actinobacteria, and Fusobacteria were dominant in all groups. The phyla Verrucomicrobia and Proteobacteria were significantly increased in the LC group compared with those in other groups, whereas the phyla Tenericutes were significantly less abundant (Figure 4A). In the HCC groups, the phylum Fusobacteria was significantly more abundant than that in the other groups. There was no significant difference in the phyla, Actinobacteria, Bacteroidetes, and Firmicutes among all of the groups.

Regarding the differences in bacterial abundance at the genus level, a total of 79 genera were observed, of which 51 showed significant differences when evaluated with the univariate method (*P* < .05 and FDR < 0.05, Table S1). Here, the TOP12 differential genera are shown in Figure 4, and other genera are presented in the Table S2. Specifically, the GM of patients with LC was enriched with *Phyllobacterium*, *Sphingomonas*, *Enterococcus*, *Erysipelatoclostridium*, and *Romboutsia* compared with the GM of the other groups, while the abundances of *Ralstonia*, *Catenibacterium*, and *Lachnospira* were decreased. The patients with HCC showed a significantly increased level of *Sarcina* compared to the other groups. The abundance of *Neisseria* was higher in hepatitis patients than that in other patients. Furthermore, the healthy people showed a higher abundance of *Mitsuokella* and a lower abundance of *Ralstonia*.

**Figure 5:**
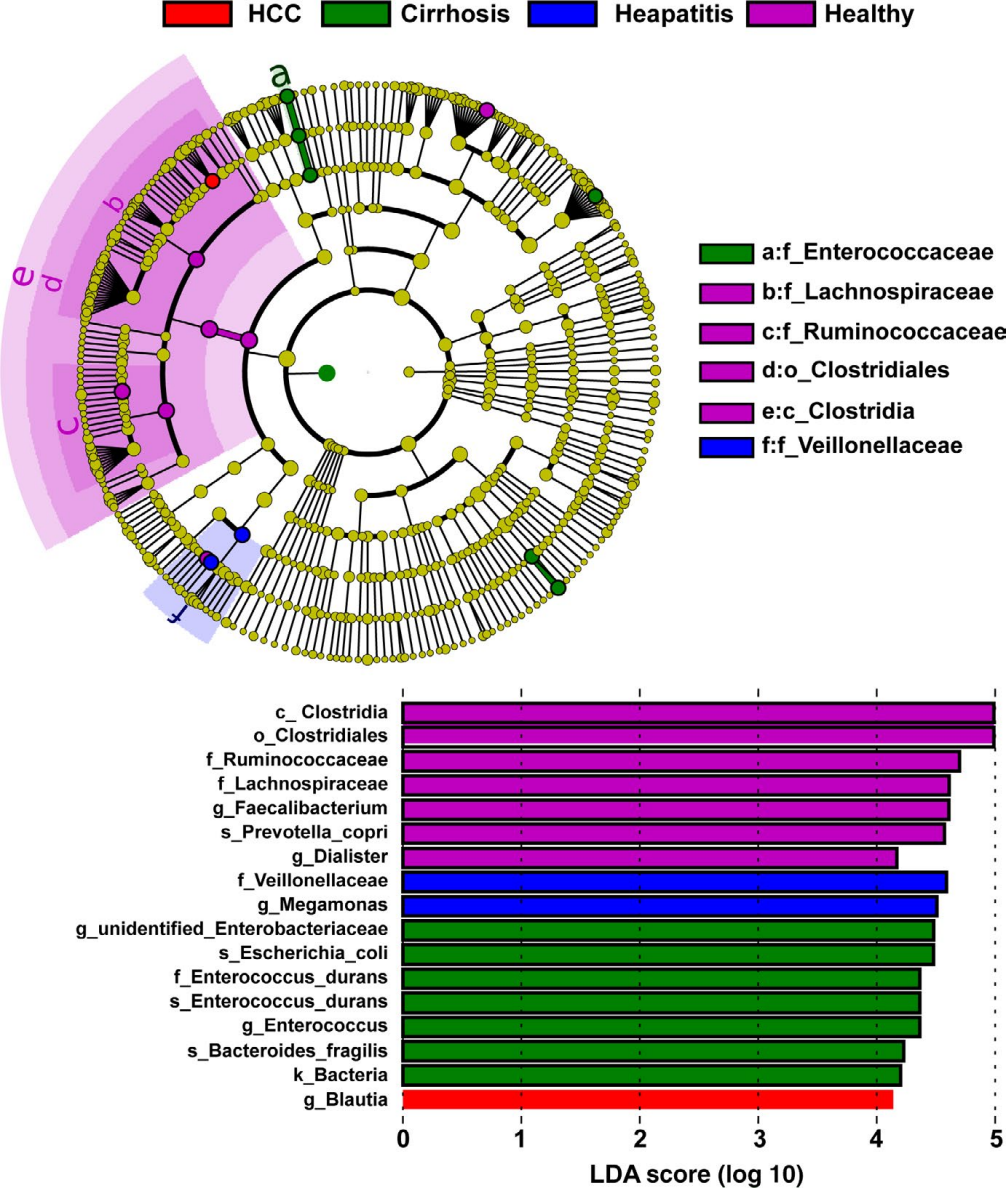
LEfSe analysis among the healthy, hepatitis, LC, and HCC groups. The differentially abundant taxa in the taxonomic tree are shown in the cladogram in different colors. The LDA scores greater than 4.0 for the significantly differentially abundant bacteria are displayed in the histogram with different colors. LC, liver cirrhosis; HCC, hepatocellular carcinoma; LEfSe, linear discriminant effect size. LDA, linear discriminant analysis

To further confirm the biological taxonomic differences among the four groups, we then performed LEfSe analysis with an LDA score ≥4.0. As shown in Figure 5, the GM of patients with LC was enriched with g_ *Enterobacteriaceae*, s_*Escherichia_coli*, f_Enterococcus_durans, s_*Enterococcus_durans*, g_*Enterococcus* and s_*Bacteroides_fragilis*. For healthy people, increased abundances of c_Clostridia, o_Clostridiales, f_Ruminococcaceae, f_Lachnospiraceae, g_*Faecalibacterium*, s_*Prevotella_copri,* and g_*Dialister* were observed. Moreover, increased abundances of f_Veillonellaceae and g_*Megamonas* were found in hepatitis patients, and only g_*Blautia* was enriched in the patients with HCC.

### 3.5 Low biodiversity of the GM in LC‐HCC

**Figure 6:**
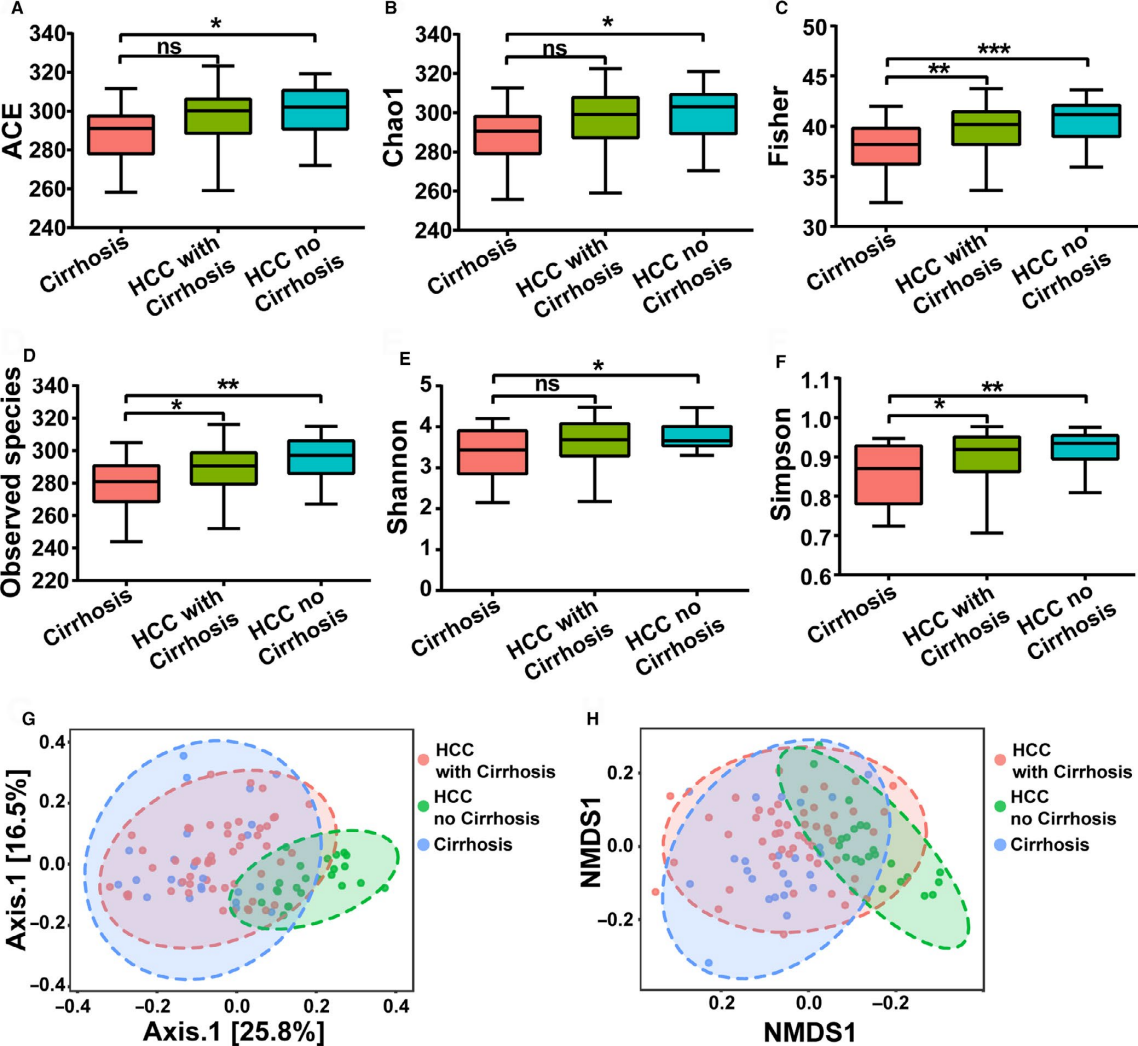
Alpha‐diversity analysis and beta‐diversity analysis among the LC, LC‐HCC, and NLC‐HCC groups. A, ACE index. B, Chao1 index. C, Fisher index. D, Observed species index. E, Shannon index. F, Simpson index. G, PCoA analysis based on the unweighted UniFrac distance matrix. H, NMDS analysis. Data are shown as the mean ± SD, *P <* *.05; **P <* *.01; ***P <* *.001; NS, nonsignificant. LC, liver cirrhosis; HCC, hepatocellular carcinoma; LC‐HCC, liver cirrhosis‐induced HCC; NLC‐HCC, nonliver cirrhosis‐induced HCC; PCoA, principal coordinates analysis; NMDS, nonmetric multidimensional scaling

Based on the results of the previous part of the experiment, we found that the diversity of the GM of HCC patients did not decrease significantly with the deterioration of liver disease progression, on the contrary, it was significantly higher than that of the LC patients. To further confirm the GM of patients with HCC, we divided 75 HCC patients into LC‐HCC (n = 52) and NLC‐HCC (n = 23) according to the presence or absence of LC in HCC patients. Subsequently, alpha‐diversity and beta‐diversity analyses between the two groups were performed and compared to the LC group. Compared to the LC group, as shown in Figure 6A‐F, the diversity of the GM showed a significant increase in the NLC‐HCC, which was estimated by the ACE index (*P *< .05), Chao1 index (*P *< .05), Fisher index (*P *< .001), Observed species index (*P *< .05), Shannon index (*P *< .05), and the Simpson index (*P *< .01). Compared with the LC group, the Fisher index, Observed species index and Simpson index in the LC‐HCC group were increased (*P *< .05), while the ACE, Chao1, and Shannon indexes showed no significant difference (*P *> .05). Similar to the alpha‐diversity analysis results, the beta‐diversity analysis results also showed that significant clustering was formed in the NLC‐HCC group, which was distinguished from the LC‐HCC and LC groups. In summary, these results demonstrated that gut microbial diversity was closely related to the presence or absence of LC in patients with HCC, rather than the HCC itself.

### 3.6 Phylum‐level and genus‐level changes in the GM community in the LC‐HCC group

**Figure 7:**
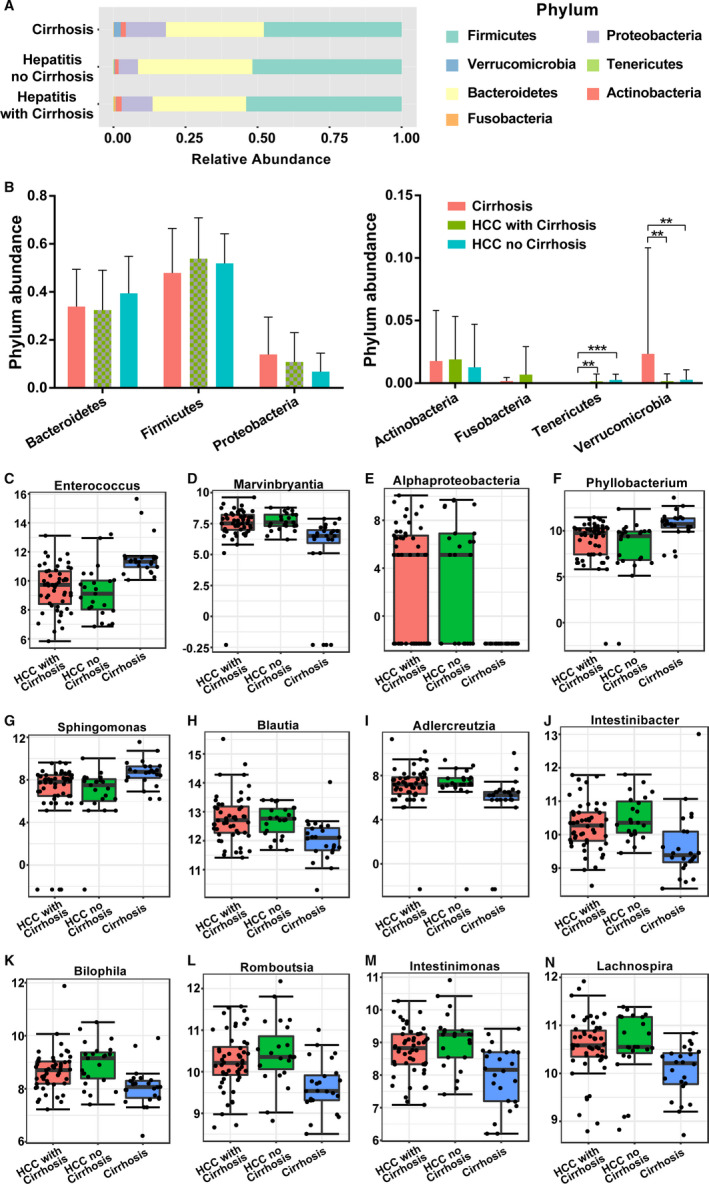
The differences in GM among the LC, LC‐HCC, and NLC‐HCC groups at the Phylum and genus levels. A,B, Composition of the GM at the phylum level. The top 12 most abundant components of the GM at the genus level are shown as (C) *Enterococcus*, (D) *Marvinbryantia*, (E) *Alphaproteobacteria*, (F) *Phyllobacterium*, (G) *Sphingomonas*, (H) *Blautia*, (I) *Adlercreutzia*, (J) *Intestinibacter*, (K) *Bilophila*, (L) *Romboutsia*, (M) *Intestinimonas,* and (N) *Lachnospira*. GM, gut microbiota; LC, liver cirrhosis; HCC, hepatocellular carcinoma; LC‐HCC, liver cirrhosis‐induced HCC; NLC‐HCC, nonliver cirrhosis‐induced HCC

A total of seven phyla including Firmicutes, Proteobacteria, Verrucomicrobia, Tenericutes, Bacteroidetes, Actinobacteria, and Fusobacteria were dominant in the LC, LC‐HCC, and NLC‐HCC groups. The phyla Fusobacteria and Proteobacteria were significantly increased in the LC group compared with those in the LC‐HCC and NLC‐HCC groups, whereas the phylum Tenericutes was significantly less abundant (Figure 7A). As for the NLC‐HCC group, Bacteroidetes was increased and Actinobacteria was decreased. Only the Firmicutes phylum was increased in the LC‐HCC group compared to that in the LC and NLC‐HCC groups.

A total of 80 genera were observed, of which 26 had significant differences evaluated with the *P* values and FDR values being both less than .05 (Table S2). In the TOP12 differential genera, *Enterococcus*, *Phyllobacterium*, *Sphingomonas*, and *Lachnospira* were significantly increased in the LC group, while *Marvinbryantia*, *Alphaproteobacteria*, *Phyllobacterium*, *Adlercreutzia*, *Bilophila*, and *Romboutsia* were significantly decreased. The abundance of *Intestinibacter* and *Intestinimonas* was higher in the NLC‐HCC group, and the abundance of *Blautia* was significantly increased in the LC‐HCC group (Figure 7B‐M).

**Figure 8:**
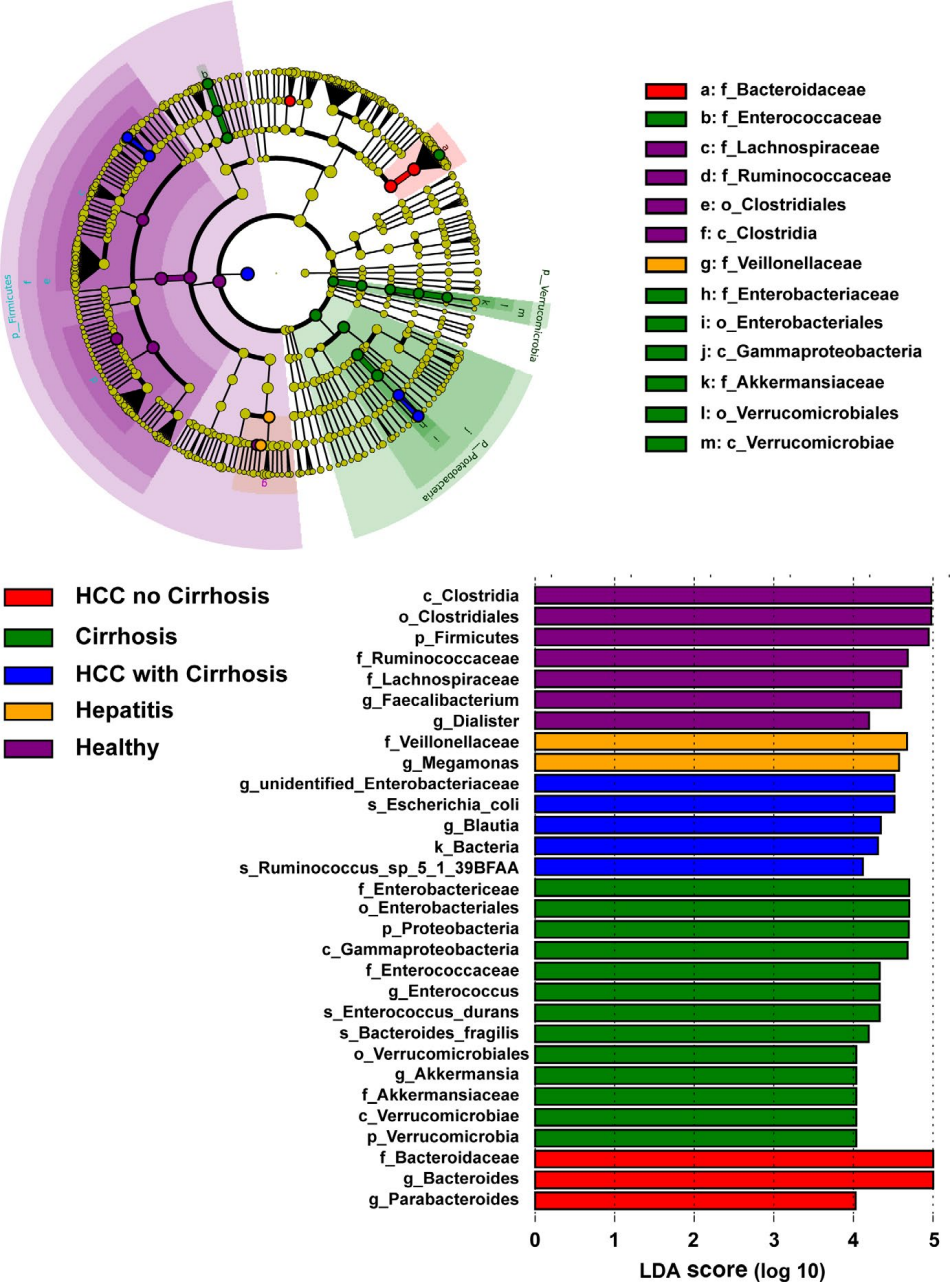
LEfSe analysis among the healthy, hepatitis, LC, LC‐HCC, and NLC‐HCC groups. The differentially abundant taxa in the taxonomic tree are shown in the cladogram in different colors. The LDA scores greater than 4.0 for the significantly differentially abundant bacteria are displayed in the histogram with different colors. LC, liver cirrhosis; HCC, hepatocellular carcinoma; LC‐HCC, liver cirrhosis‐induced HCC; NLC‐HCC, nonliver cirrhosis‐induced HCC; LEfSe, linear discriminant effect size; LDA, linear discriminant analysis

LEfSe analysis with an LDA score ≥4.0 was also conducted among the healthy, hepatitis, LC, LC‐HCC, and NLC‐HCC groups. As shown in Figure 8, the GM of the LC‐HCC patients was enriched with g_*Enterobacteriaceae*, s_*Escherichia*_*coli*, g_*Blautia,* and s_*Ruminococcus*_sp_5_1_39BFAA. However, increased abundances of f_Bacteroidaceae, g_*Bacteroidaceae,* and g_*Prabacteroides* were observed in the NLC‐HCC group.

### 3.7 Association between environmental factors of HCC and GM

**Figure 9:**
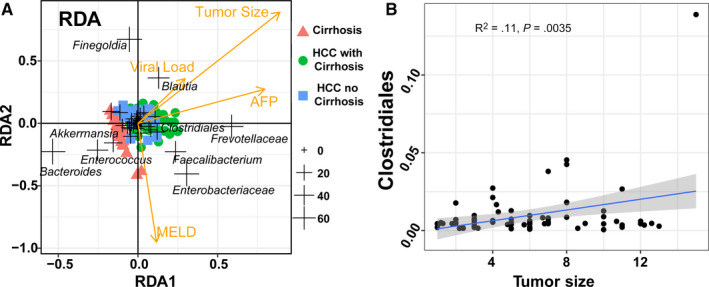
The general analysis of the association between environmental factors and GM. A, Redundancy analysis of the microbiota of HCC and LC with marked genera displayed. B, The correlation results between the GM content and tumor size. Only *Clostridiates* was found to be tightly correlated with tumor size in the general HCC group (*P* = .0035, *R* ^2^ = .11). AFP, alpha‐fetoprotein; GM, gut microbiota; LC, liver cirrhosis; HCC, hepatocellular carcinoma; LC‐HCC, liver cirrhosis‐induced HCC; NLC‐HCC, nonliver cirrhosis‐induced HCC; RDA, redundancy analysis

Redundancy analysis (RDA) was utilized here to display the environmental factors related to the pathogenesis of HCC and LC. As displayed in Figure 9A, all samples from HCC or LC patients were nearly randomly and evenly distributed on both sides of the potential environmental risks, which indicated that, in the present study, the difference in GM structure was caused by the nature of the diseases, not by the clinical characteristics. In addition, we also observed nine genera markedly related to diseases progression. Therefore, to further explore the correlation between the most significant factor of diseases, tumor size, and intestinal bacteria, a correlation analysis was performed. As shown in Figure 9B, only *Clostridiates* was generally observed to be positively related to the tumor size of HCC.

**Figure 10:**
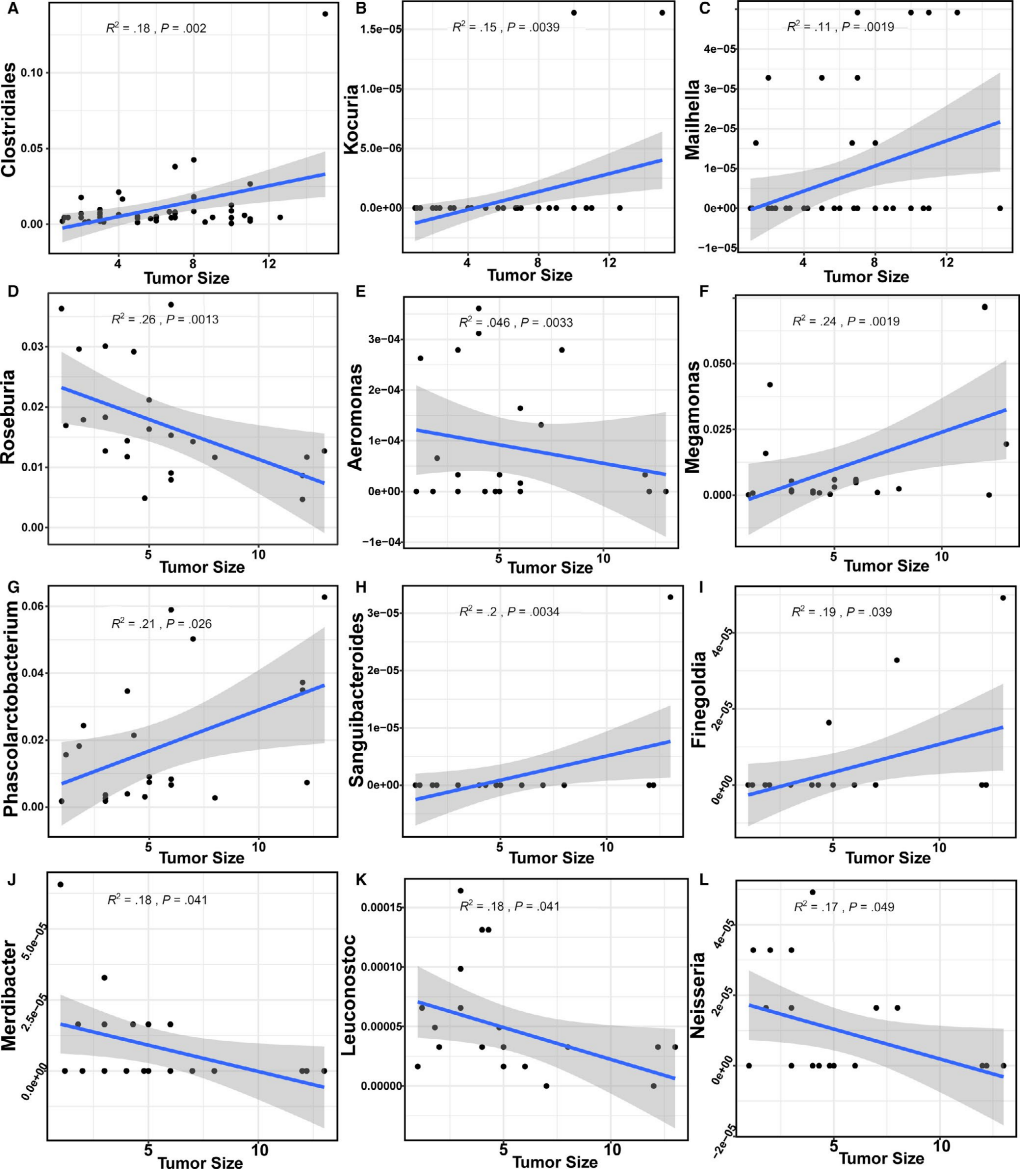
The genera in LC‐HCC and NLC‐HCC, that were correlated with tumor size. A‐C, The genera identified in LC‐HCC. D‐L, The genera identified in NLC‐HCC. The confidence interval was *P* < .05 and *R* ^2^ > .1. LC, liver cirrhosis; HCC, hepatocellular carcinoma; LC‐HCC, liver cirrhosis‐induced HCC; NLC‐HCC, nonliver cirrhosis‐induced HCC

In addition, we specifically investigated the correlation between tumor size and GM content in LC‐HCC and NLC‐HCC. Three genera (as shown in Figure 10A‐C) and nine genera (in Figure 10D‐L) were reported to be significantly correlated with the tumor size of LC‐HCC and NLC‐HCC, respectively.

**Figure 11:**
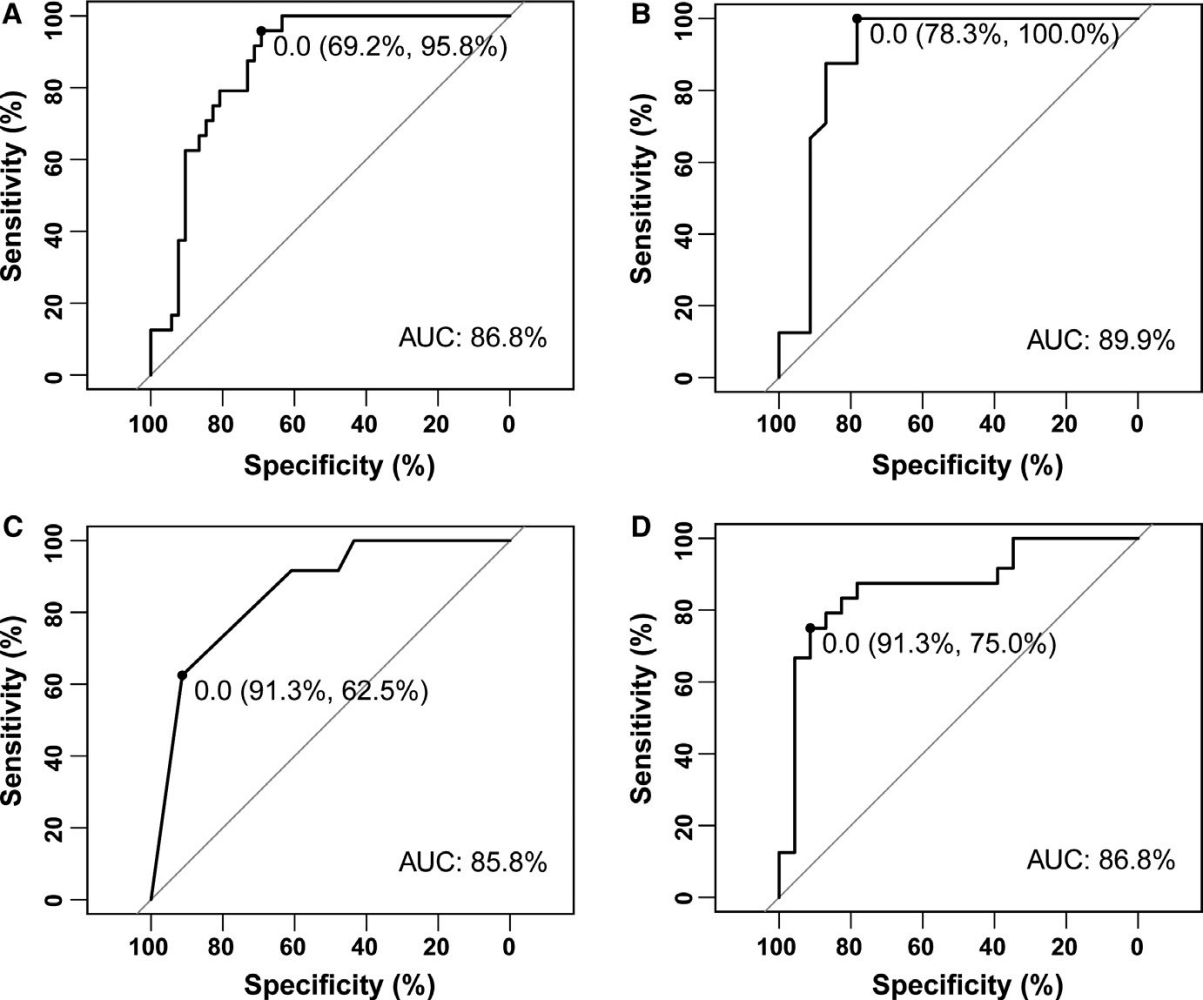
Biomarkers among LC, LC‐HCC, and NLC‐HCC with high accuracy (AUC > 0.85). *Enterococcus* could be used as a biomarker between LC and LC‐HCC (A) and between LC and NLC‐HCC (B). *Limnobacter* (C) and *Phyllobacterium* (D) could also be used as biomarkers between LC and NLC‐HCC, with higher relative abundance levels in NLC‐HCC and LC, respectively. LC, liver cirrhosis; HCC, hepatocellular carcinoma; LC‐HCC, liver cirrhosis‐induced HCC; NLC‐HCC, nonliver cirrhosis‐induced HCC

Herein, we also mined potential biomarkers in the GM of LC, LC‐HCC, and NLC‐HCC. As displayed in Figure 11, one biomarker and three biomarkers (AUC > 0.85) were identified in LC‐HCC and NLC‐HCC, respectively. There was no significant biomarker between HCC and LC (AUC < 0.7).

### 3.8 Relationship between HCC etiology and GM

**Figure 12:**
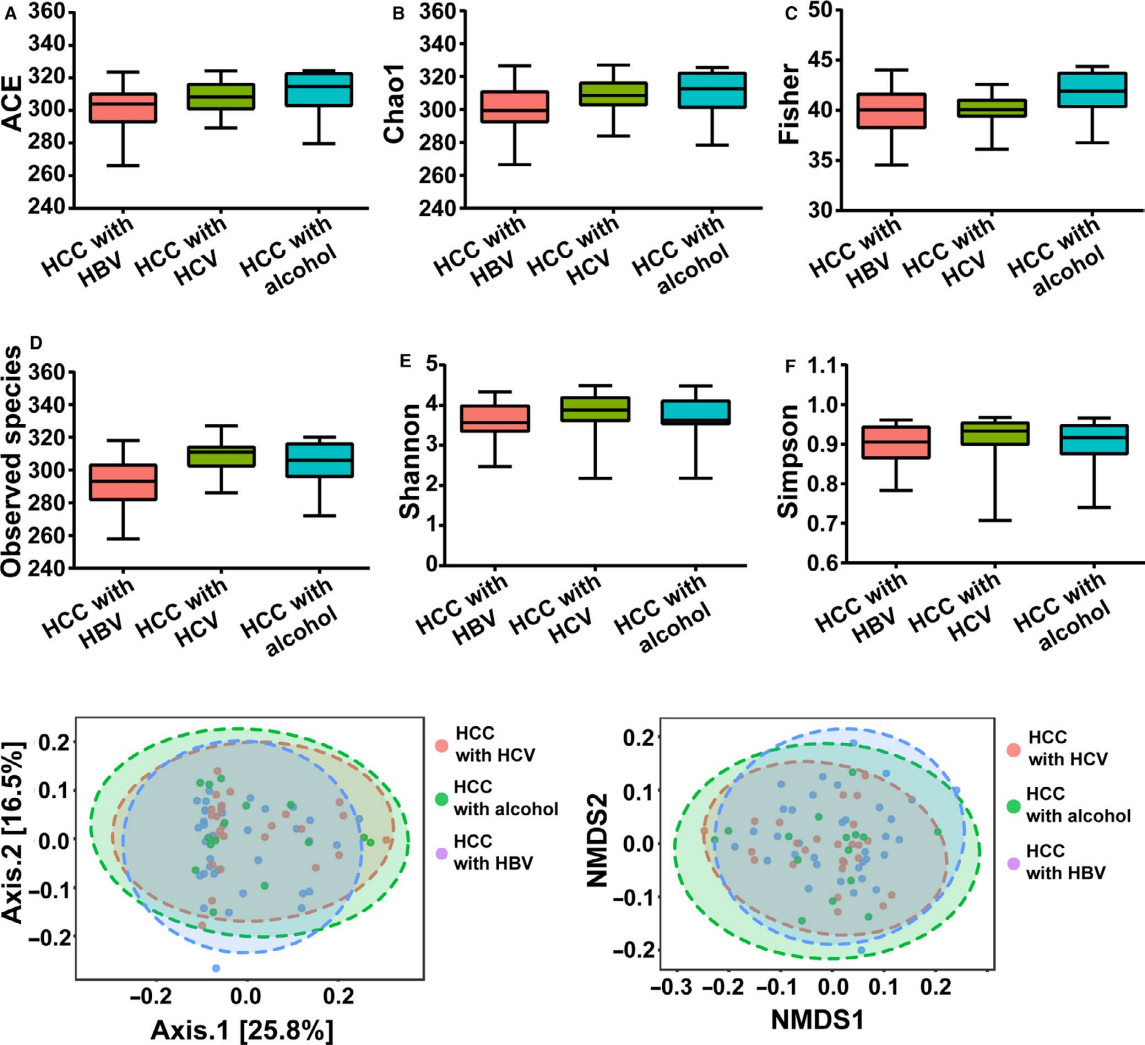
Alpha‐diversity analysis and beta‐diversity analysis among HCC with HBV, HCV, or alcohol groups. A, ACE index. B, Chao1 index. C, Fisher index. D, Observed species index. E, Shannon index. F, Simpson index. G, PCOA analysis based on unweighted UniFrac distance matrix (H) NMDS analysis. Data are shown as mean ± SD, *P <* *.05; **P <* *.01; ***P <* *.001. HCC, hepatocellular carcinoma; HBV, hepatitis B virus; HCV, hepatitis C virus; PCoA, principal coordinates analysis; NMDS, nonmetric multidimensional scaling

**Figure 13:**
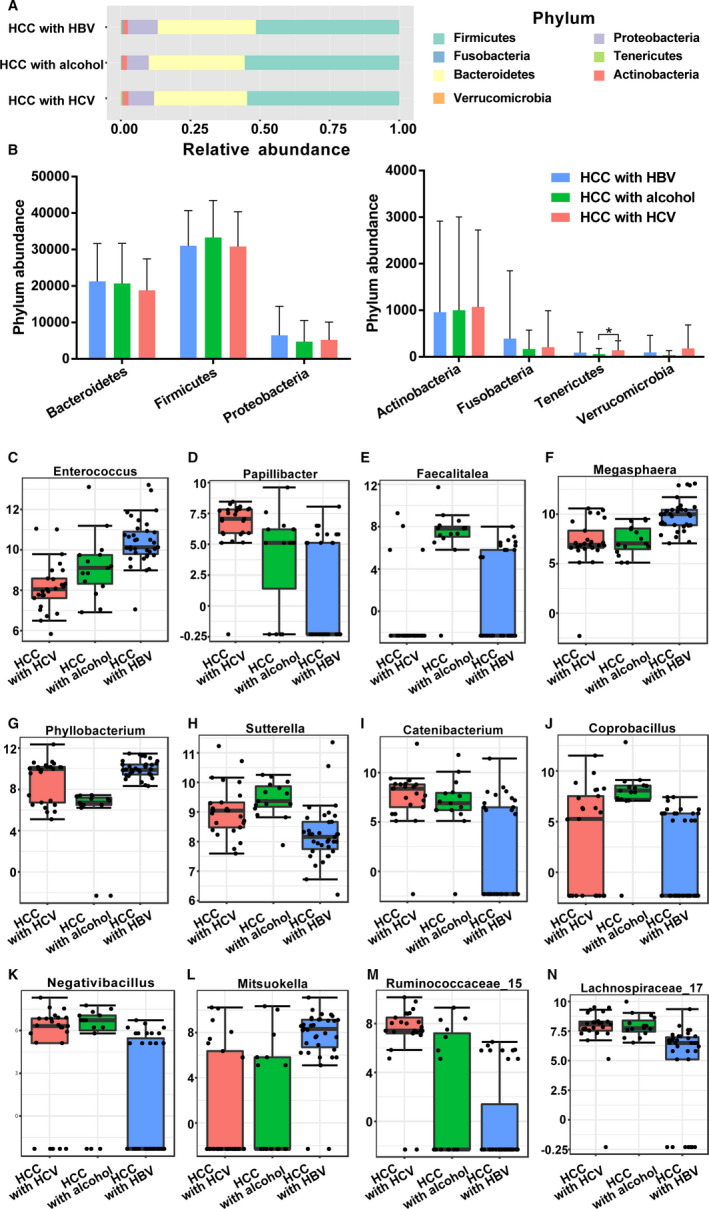
The differences of gut microbiota among three groups at phylum level and genus level. A,B Composition of gut microbiota at phylum level. The top 12 taxonomies of gut microbiota at genus level were shown as (C) *Enterococcus*, (D) *Papillibacter*, (E) *Faecalitalea*, (F) *Megasphaera*, (G) *Phyllobacterium*, (H) *Sutterella*, (I) *Catenibacterium*, (J) *Coprobacillus*, (K) *Negativibacillus*, (L) *Mitsuokella*, (M) *Ruminococcaceae*, and (N) *Lachnospiraceae*. HCC, hepatocellular carcinoma; HBV, hepatitis B virus; HCV, hepatitis C virus

To further explore GM according to the different etiologies of HCC, we categorized HCC patients into HBV‐HCC, HCV‐HCC, and ALD‐HCC. Alpha‐diversity and beta‐diversity analyses showed that there were no significant differences among them (Figure 12). Although 18 differentiated genera were found, there were no significant differences at the phylum level (as shown in Figure 13; Table S3). Taken together, these results indicated that the different causes of HCC were not the direct determinant factors for the dysbiosis or alteration of the GM in HCC.

## 4 DISCUSSION

In this study, we demonstrated that the fecal microbial diversity was significantly decreased in the LC group, as 3 phyla and 27 genera had significant differences in the LC group vs the healthy, hepatitis, and HCC groups. Interestingly, the diversity was significantly increased from LC to HCC. In order to further confirm the roles of the GM in the pathogenesis of HCC, we identified the fecal microbiota of LC‐HCC and NLC‐HCC patients. We found that the gut microbial diversity of NLC‐HCC was significantly increased compared to that of LC, while the diversity of LC‐HCC had no differences from LC. Furthermore, we found that gut microbial dysbiosis was not associated with the different causes of HCC, such as HBV, HCV, or ALD. This is the first study to characterize the GM in HCC patients from Northeast China, and the results underscore that LC might be the main cause of gut microbial dysbiosis in patients with HCC.

GM plays crucial roles in the pathogenesis of diverse liver diseases, ranging from hepatitis, to LC to HCC.^26^ Intestinal microorganisms could be transferred to the liver through the gut‐liver axis, which initiated the inflammatory cascades and promoted the progression from severe LC to HCC.^27^ Despite the consensus on the association between the GM and liver diseases, these studies have focused only on the relationship between the GM and a specific liver disease.^28^, ^29^, ^30^ In our study, we characterized and compared the alternation of GM among healthy, hepatitis, LC, and HCC patients from Northeast China for the first time. We demonstrated that the gut microbial diversity was significantly decreased in patients with LC, which is consistent with previous studies.^31^, ^32^ There are significant differences in four phyla (Verrucomicrobia, Proteobacteria Fusobacteria, and Tenericutes) in LC patients, similar to previous research results.^21^ At the genus level of top 20, seven genera such as *Phyllobacterium*, *Sphingomonas*, and *Enterococcus* were significantly enriched in the LC group, and seven genera such as *Lachnospira*, *Catenibacterium*, and *Marvinbryantia* were decreased in the LC group. The genera *Veillonella* and *Clostridium*, which have been reported to be enriched in LC patients, were also found to be significantly elevated in our study, but they were not ranked in the top 20 most abundant genera, and we did not find a decrease in *Eubacterium* and *Alistipes*.^21^ We think this might be due to the difference in dietary habits, because it has been reported that dietary habits could rapidly and reproducibly alters the human gut microbiome and modulates the risk of hospitalization in patients with cirrhosis.^33^, ^34^

It is worth nothing that the diversity of HCC patients was not significantly different compared with the diversity in hepatitis and healthy. However, the fecal microbial diversity of HCC was significantly increased compared to the diversity of LC, which is similar to the results of Ren's research, and they also found that gut microbial diversity was increased from LC to early HCC.^25^ These results indicated that the GM has undergone a significant shift from LC to HCC, while it could not indicate that there were no changes in the bacterial structure of the HCC patients when compared to healthy subjects. Indeed, we found that the phyla Bacteroidetes and Fusobacteria were significantly enriched in HCC patients compared to those in healthy patients. In the top 20 most abundant genera, five of them such as *Neisseria*, *Mitsuokella*, and *Butyricicoccus* were the most enriched in HCC. Thus, the higher abundance or diversity of the GM in HCC patients is not a sign of healthy GM, perhaps because the various harmful bacteria or archaea were excessively reproducing. The same point of view was also raised in some other studies.^20^, ^25^

Because the gut microbial diversity of HCC was significantly higher than that of LC, we then characterized and compared the fecal microbials between LC‐HCC and NLC‐HCC. We demonstrated that the diversity of the GM showed a significant increase in NLC‐HCC compared to that in LC, while there were no significant differences observed between LC‐HCC and LC. Similar results were also found in these studies.^20^, ^25^ These results suggest that the presence of LC may be the major cause of gut microbial dysbiosis in HCC patients. Furthermore, the RDA results showed that 13 genera were associated with the tumor size of HCC. Four could be used as biomarkers among LC, LC‐HCC, and NLC‐HCC. These findings will contribute to the early diagnosis of HCC. Moreover, we also evaluated the effects of different pathogenic factors of HCC, such as HBV, HCV, and ALD, on the GM. A number of studies have confirmed that HBV‐, HCV‐, or ALD‐induced liver cancer can cause disorders of the GM in HCC patients.^35^, ^36^, ^37^ A recent study showed a differential abundance of bacteria between HBV‐HCC and non‐HBV non‐HCV‐related HCC patients.^38^ However, our results showed that there was no significant difference in the microbial structure among the three groups as a whole, although the individual genera had obvious differences. We thought that geographic differences may be the main reason for the different results. As shown in previous studies, the changes in the characteristics of the GM were closely related to the location of the host.^39^

GM plays vital roles in maintaining the health of the body, including the synthesis of beneficial substances such as vitamin K,^40^ maintaining intestinal integrity by producing short‐chain fatty acids,^41^ and competing against pathogenic microorganisms.^42^ In our study, we found that the abundance of *Clostridium*, *Ruminococcus*, and *Coprococcus* was significantly reduced in LC and HCC patients, with a higher decline in LC‐HCC. Those genera have been reported to have the ability to synthesize short‐chain fatty acids to maintain intestinal homeostasis.^43^ Short‐chain fatty acids mainly include acetate, propionate, and butyrate. Acetate is mainly used for lipogenesis, propionate is mainly consumed for gluconeogenesis, and butyrate is the preferred source of energy for colon cells.^44^ Increasing evidence indicates that these small molecules play key roles in maintaining the intestinal immune system, such as improving intestinal barrier function,^45^ inhibiting fat accumulation,^46^ exhibiting anti‐inflammatory effects, and protecting the host against colon diseases.^47^ Some universally produced butyrate bacteria have been shown to be depleted in various diseases such as early HCC, colorectal cancer, and type 2 diabetes.^25^, ^48^ In particular, *Faecalibacterium prausnitzii* has been used as a probiotic to treat liver diseases because it can produce large amounts of butyrate and has anti‐inflammatory effects.^49^ It is generally believed that *Lactobacillus* and *Bifidobacterium* mainly contain beneficial bacteria that are beneficial to the formation of the body's immune system and inhibit the growth of other harmful bacteria.^50^ Indeed, we found that the abundance of *Bifidobacterium* was significantly reduced in LC but not HCC *Lactobacillus* was also decreased in LC patients, while there was no significant difference. Moreover, *Akkermansia*, as a new anticancer star bacterium, has been proven not only to inhibit the occurrence of obesity but also to promote intestinal barrier function and delay the progression of cancer.^51^ However, in our study, we did not find significant differences at the genus level among the four groups, but it showed a downward trend in LC and HCC patients. Taken together, the reduction in beneficial bacteria, especially those producing butyrate, may be the main cause of progression of HCC or LC.

Moreover, we found that a large number of harmful genera, such as *Neisseria*, *Peptostreptococcus*, *Enterobacteriaceae*, and *Veillonella* were significantly decreased in LC‐HCC, while the abundance of these genera was lower in LC patients. These genera are taxonomically assigned species of buccal origin, suggesting an invasion of the gut from the mouth in LC.^25^ Furthermore, most of them can produce LPS, which causes the body's inflammatory cascade and aggravates the deterioration of liver diseases.^48^ For example, *Enterococcus* has been shown to produce polysaccharide A and LPS, which in turn facilitates the translocation of LPS into liver cells.^52^ Intestinal permeability is increased in LC patients, which exposes the liver to gut‐derived harmful bacteria. The accumulation of LPS produced by these harmful bacteria generates liver inflammatory reactions through TLR4 to provide an environment for HCC development.^53^ The complex pro‐inflammatory network could promote the secretion of inflammatory factors, the damage of hepatocytes, and the infiltration of monocytes, which are known to promote HCC progression by modulating immune responses.^54^ We thought this may be responsible for the significant reduction in gut microbial diversity in LC‐HCC patients compared to NLC‐HCC patients. Taken together, butyrate‐producing genera were decreased while LPS‐producing genera were increased in LC‐HCC patients. Therefore, relying on only one or two probiotics may have significant limitations in the treatment of liver disease, and reshaping the healthy GM through fecal microbiota transplantation, might be the most effective approach to treat liver disease, especially for LC‐HCC patients.^55^

## 5 CONCLUSIONS

Our results suggest that butyrate‐producing genera were decreased, while LPS‐producing genera were increased in LC‐HCC patients. We demonstrated that gut microbial dysbiosis in patients with HCC is associated with LC but not HBV, HCV, or ALD. We also found that four biomarkers could be used for the precise diagnosis of HCC. This study opens an avenue to the development of novel probiotics, which might help combat the aggravation of liver diseases. Furthermore, further studies of gut microbial dysbiosis may achieve early diagnosis and new therapeutic approaches for HCC patients.

## 6 TRIAL REGISTRATION

The Study on microbiome: the relationship between primary carcinoma of the liver and gut microbiome, ChiCTR‐ROC‐16010189. Registered 19 December 2016, http://www.chictr.org.cn/showproj.aspx?proj=17374.

## CONFLICT OF INTEREST

The authors declare that they have no competing interests.

## AUTHOR CONTRIBUTIONS

FW and RZ conceived and designed the study project. RZ, GW, ZP, NR, YG, and XG did experiments and performed analysis. YY, XZ, HP, and JZ collected fecal samples and organized clinical data. RZ and GW wrote the original manuscript. FW and ZP reviewed and finalized the manuscript. All authors read and approved the final manuscript.

## ETHICS APPROVAL AND CONSENT TO PARTICIPATE

This study was performed in accordance with the Helsinki Declaration and Rules of Good Clinical Practice. It was approved by the First Hospital of Jilin University Ethics Committee (2017‐342) and registered in Clinical Registry Platform (Registry ID: ChiCTR‐ROC‐16010189). All participants signed written informed consent on enrolment.

## Supporting information

Fig S1

Fig S2

Fig S3

Fig S4

Fig S5

Fig S6

Fig S7

Fig S8

Table S1‐S3

## Data Availability

The sequencing data have been deposited in the SRA database of NCBI under the Bio Project: PRJNA54057.
