# Supplementary figures and images for: Liver cirrhosis contributes to the disorder of gut microbiota in patients with hepatocellular carcinoma
Source: Cancer Med. 2020 Apr 12;9(12):4232–50. doi: 10.1002/cam4.3045 (PMC7300425; doi:10.1002/cam4.3045)

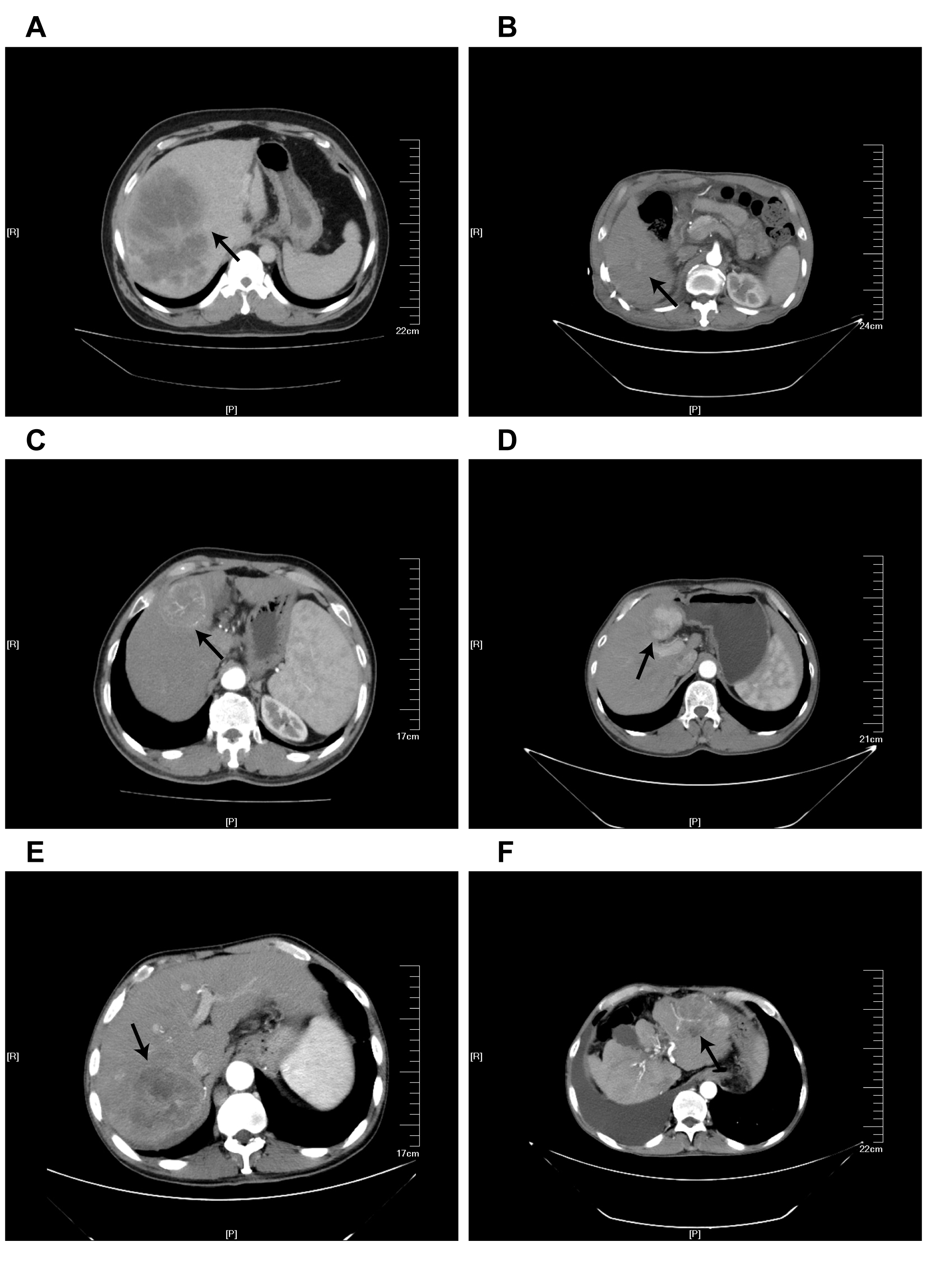

Supplement: Supplementary file 1 — Fig S1 [file CAM4-9-4232-s001.tif]

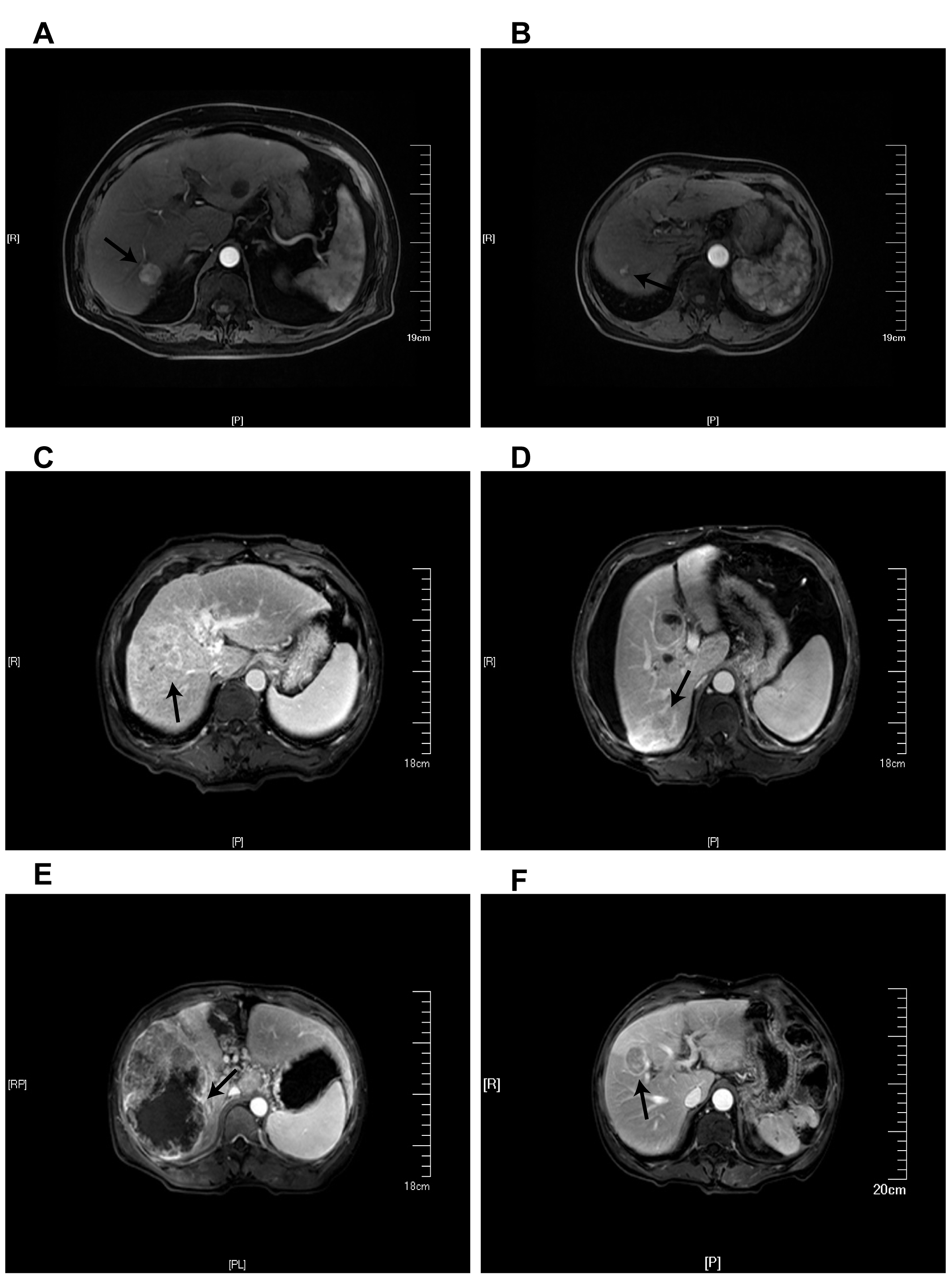

Supplement: Supplementary file 2 — Fig S2 [file CAM4-9-4232-s002.tif]

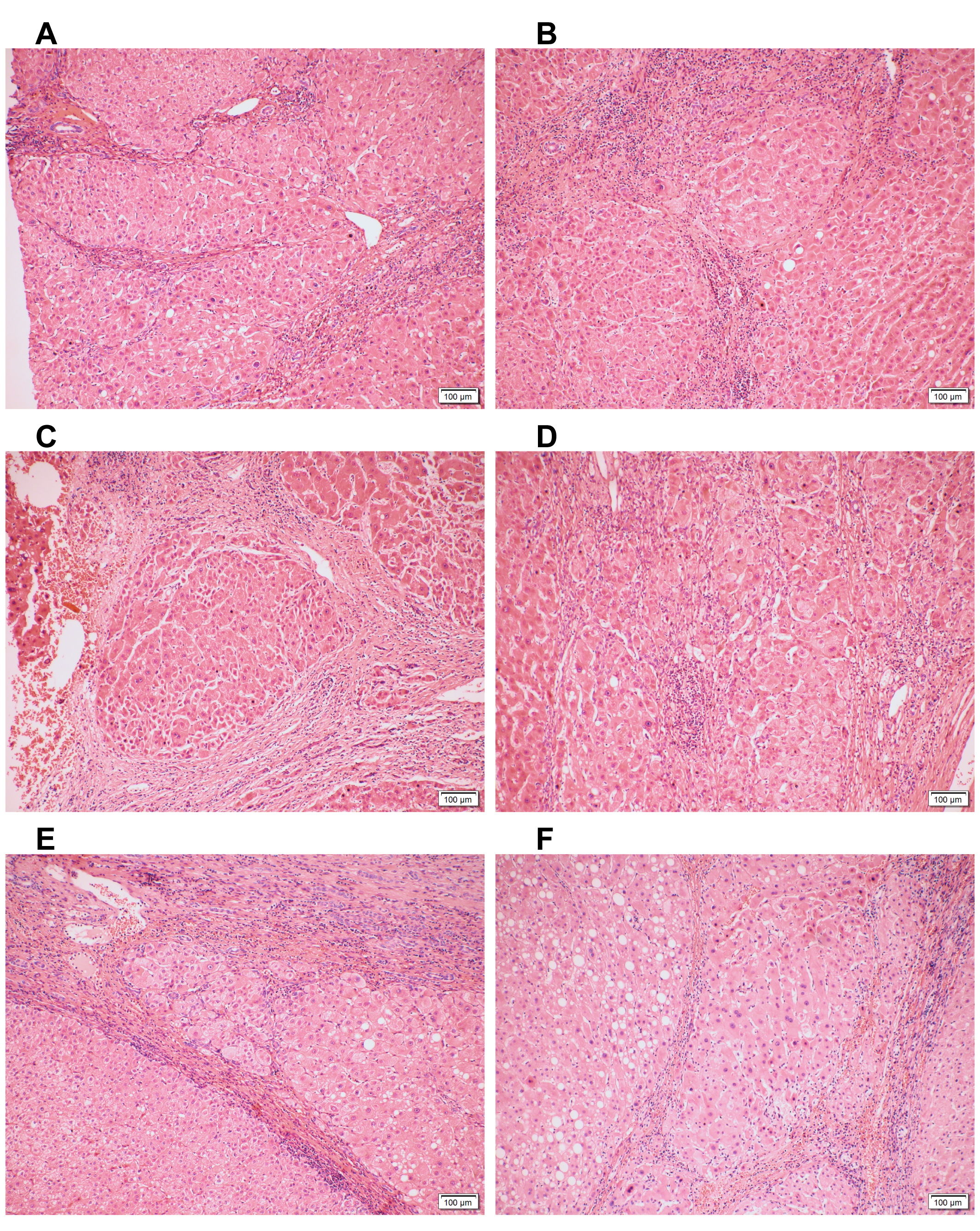

Supplement: Supplementary file 3 — Fig S3 [file CAM4-9-4232-s003.tif]

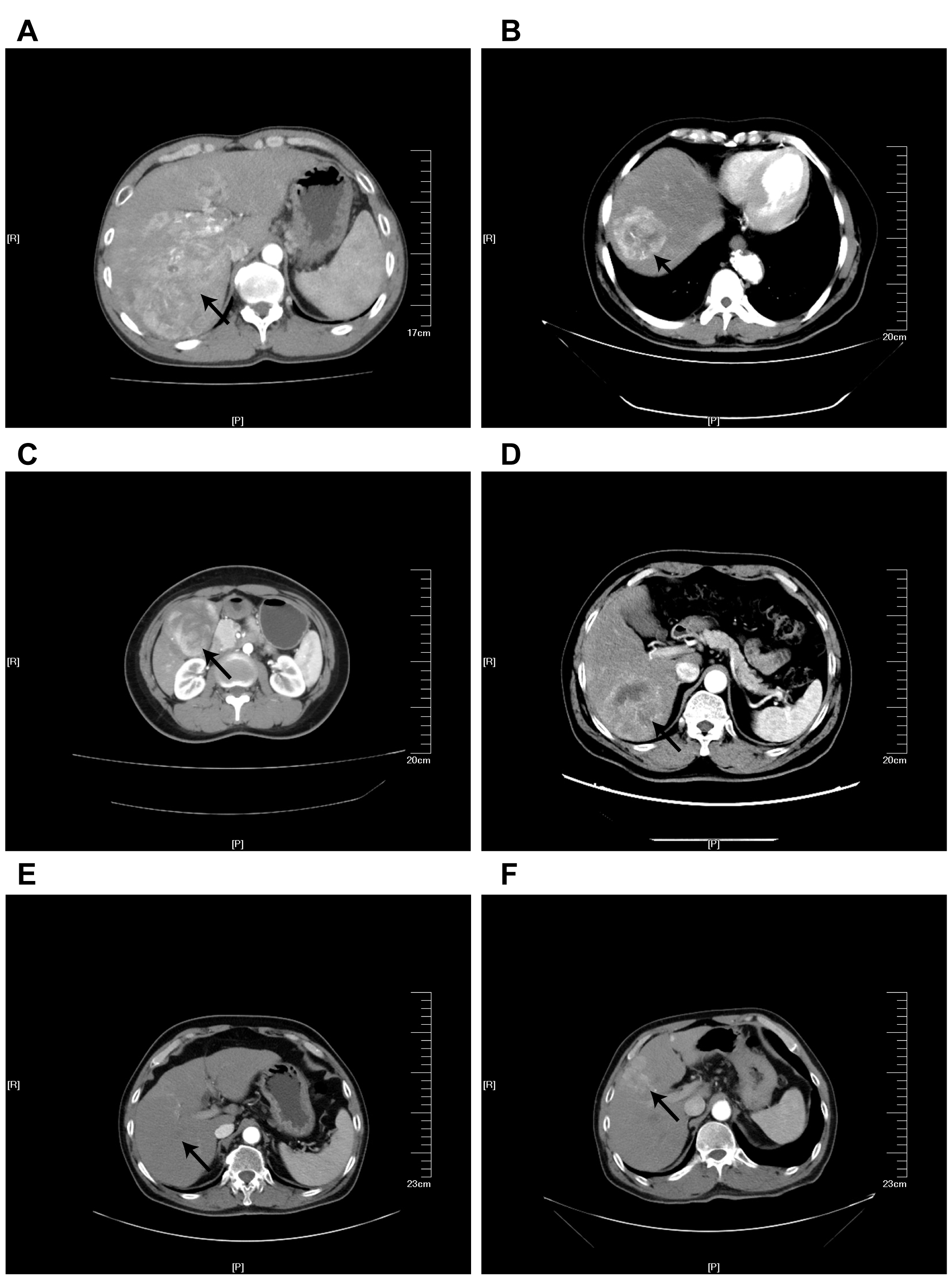

Supplement: Supplementary file 4 — Fig S4 [file CAM4-9-4232-s004.tif]

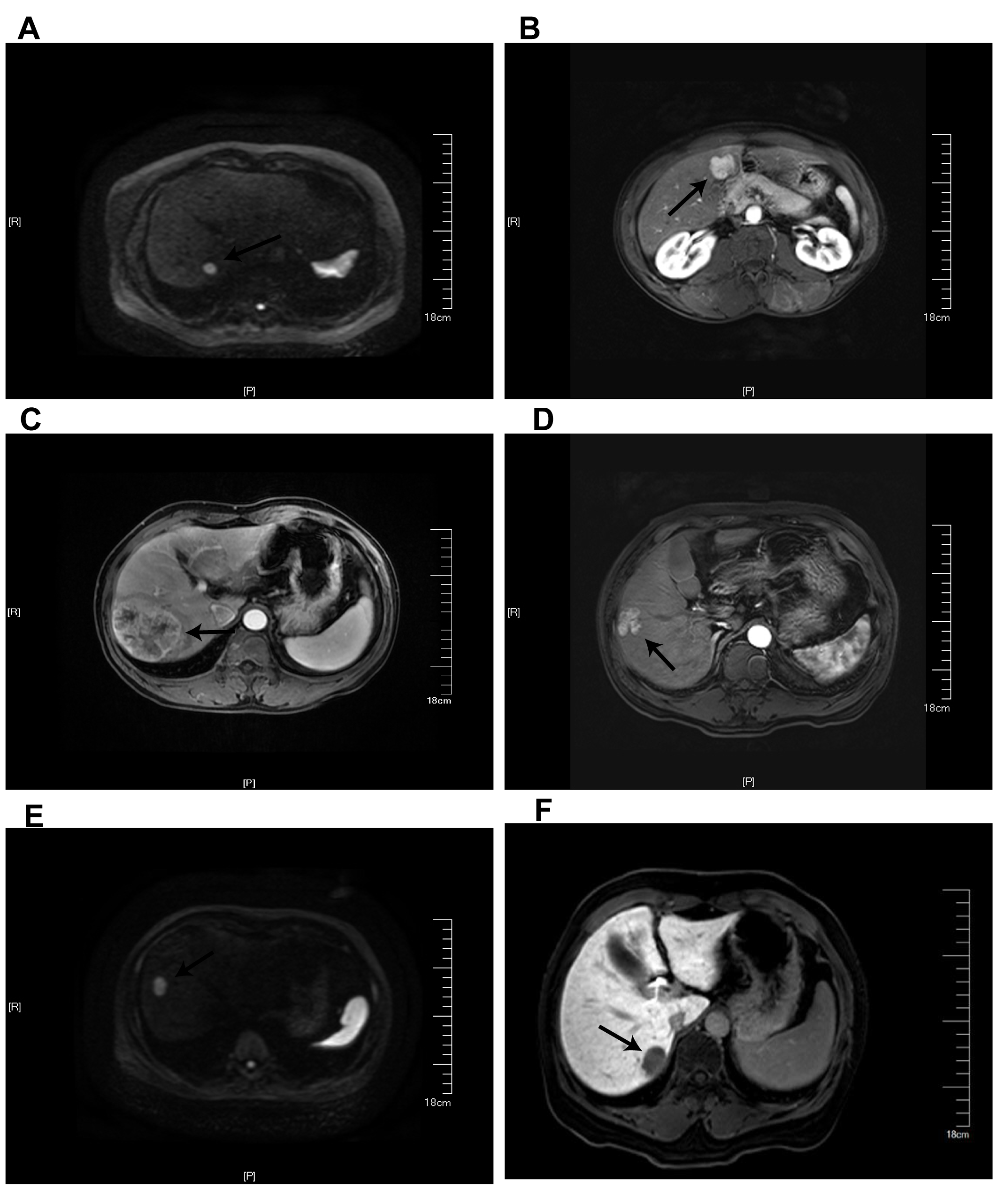

Supplement: Supplementary file 5 — Fig S5 [file CAM4-9-4232-s005.tif]

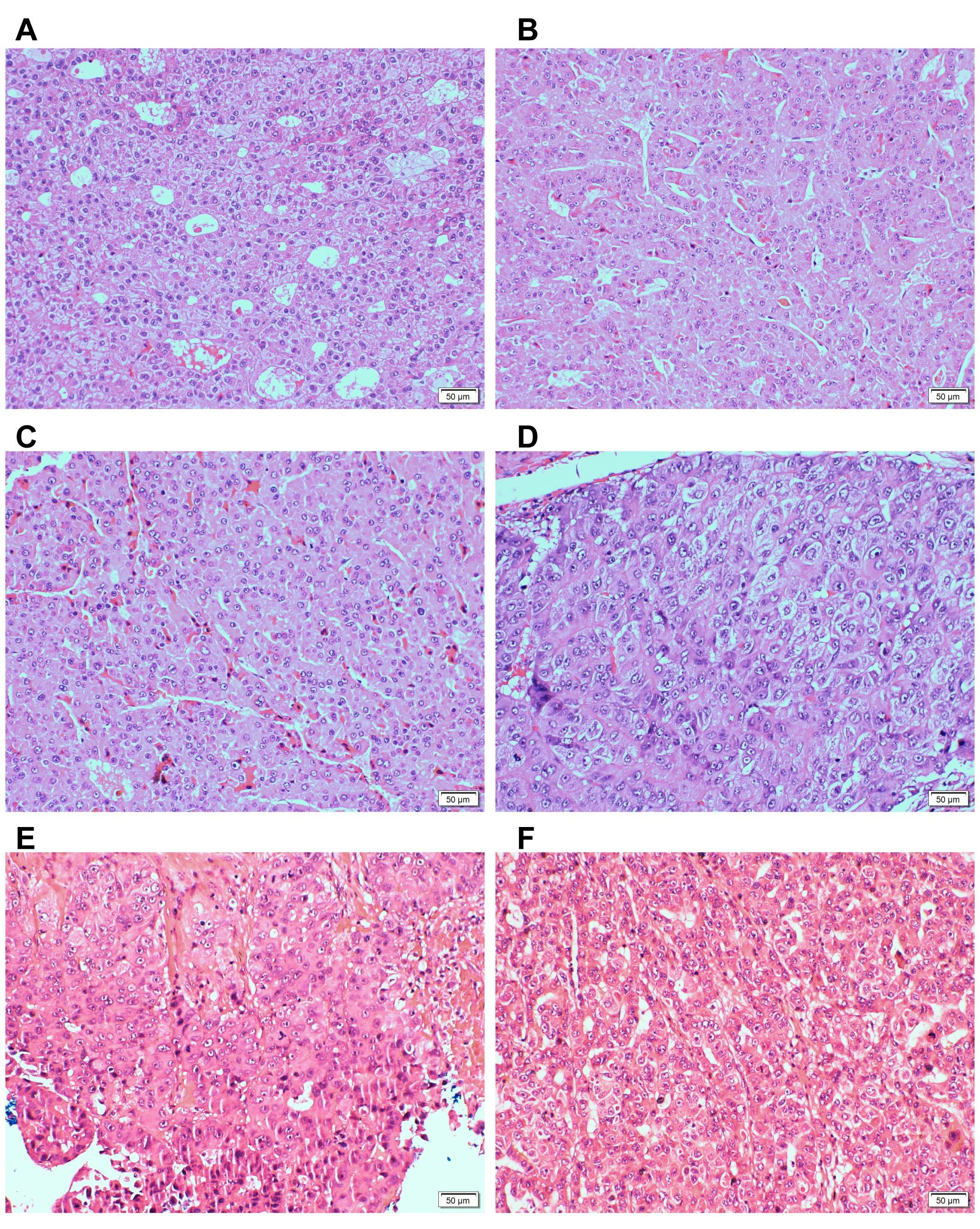

Supplement: Supplementary file 6 — Fig S6 [file CAM4-9-4232-s006.tif]

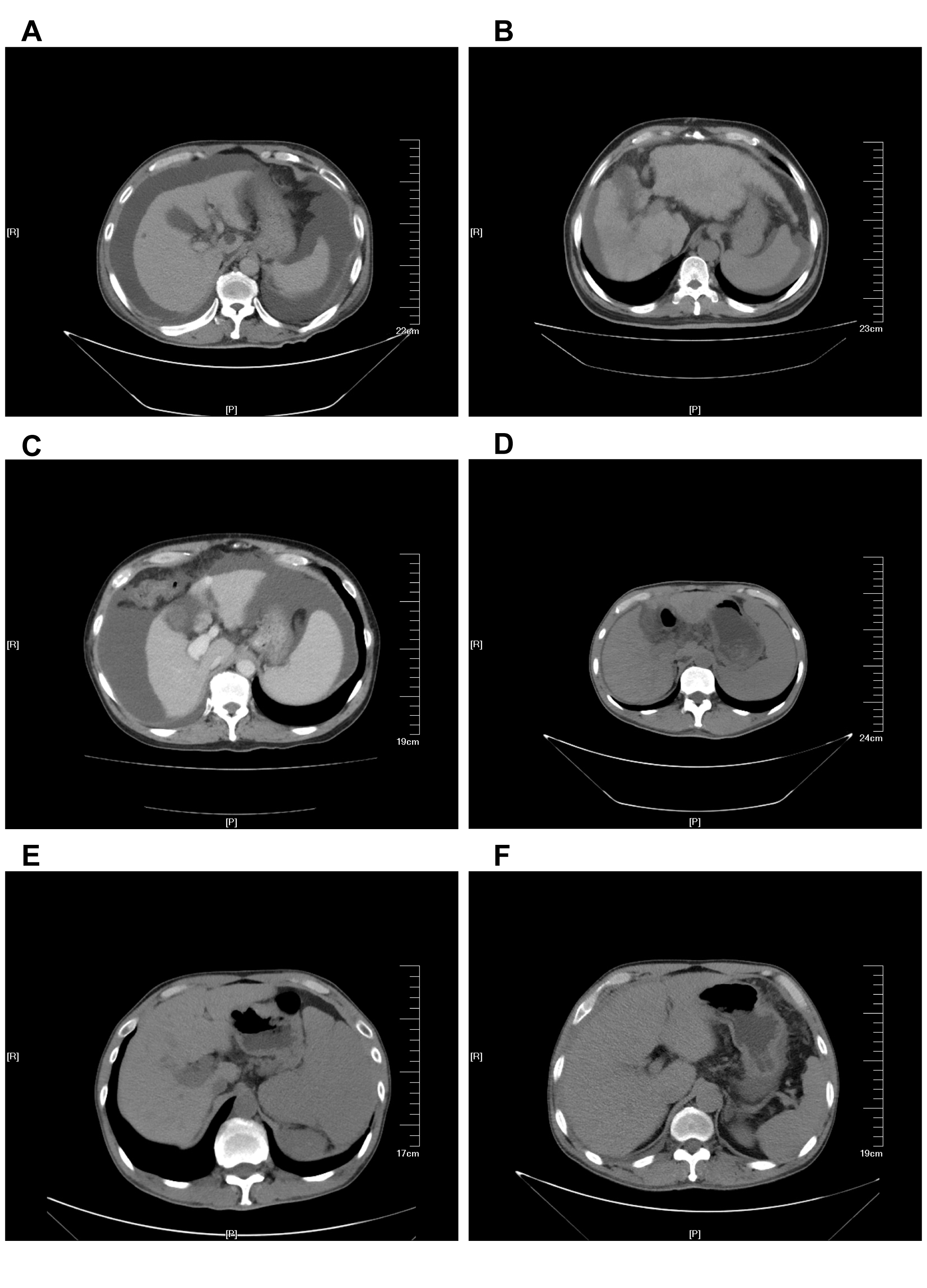

Supplement: Supplementary file 7 — Fig S7 [file CAM4-9-4232-s007.tif]

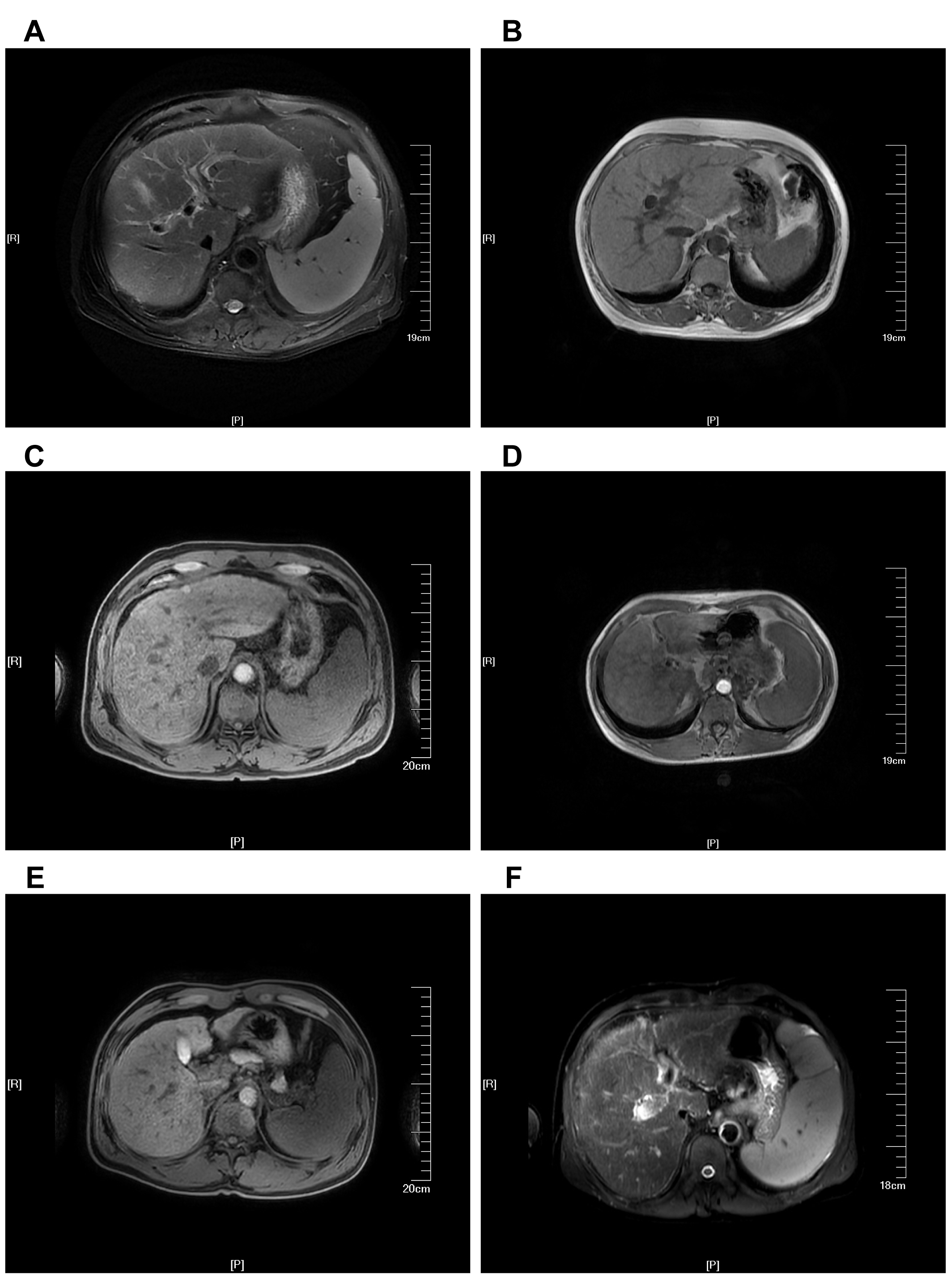

Supplement: Supplementary file 8 — Fig S8 [file CAM4-9-4232-s008.tif]
