# Supplementary material for: Liver cirrhosis contributes to the disorder of gut microbiota in patients with hepatocellular carcinoma
Source: Cancer Med. 2020 Apr 12;9(12):4232–50. doi: 10.1002/cam4.3045 (PMC7300425; doi:10.1002/cam4.3045)
Supplement: Supplementary file 9 — Table S1‐S3 [file CAM4-9-4232-s009.docx]

***Supplementary Materials***

**Liver Cirrhosis Contributes to the Disorder of Gut Microbiota in the Patients with Hepatocellular Carcinoma**

**Supplementary Figures Legends**

**Figure S1. CT diagnosis results of LC-HCC patients.** The black arrowheads indicate the location of the tumor in the liver.

**Figure S2. MR diagnosis results of LC-HCC patients.** The black arrowheads indicate the location of the tumor in the liver.

**Figure S3. The results of HE staining in pathological sections of LC-HCC patients.**

**Figure S4. CT diagnosis results of NLC-HCC patients.** The black arrowheads indicate the location of the tumor in the liver.

**Figure S5. MR diagnosis results of NLC-HCC patients.** The black arrowheads indicate the location of the tumor in the liver.

**Figure S6. The results of HE staining in pathological sections of NLC-HCC patients.**

**Figure S7. CT diagnosis results of LC patients.**

**Figure S8. MR diagnosis results of LC patients.**

**Supplementary Tables**

**Table S1**

**Table S1. Clinical phenotype information of all enrolled individuals**

| **Group** | **Gender** | **Age**  **(year)** | **BMI**  **(kg/m2)** | **AFP**  **(0-20 ng/mL)** | **Tumour size (cm)** | **Child-Pugh** | **ALT**  **(9-50 U/L)** | **AST**  **(15-40 U/L)** |
| --- | --- | --- | --- | --- | --- | --- | --- | --- |
| Healthy | M | 58 | 25.4 | 3.2 | — | — | 13 | 16 |
| Healthy | F | 62 | 21.2 | 1.6 | — | — | 42 | 23 |
| Healthy | F | 69 | 20.8 | 2.7 | — | — | 39 | 18 |
| Healthy | M | 48 | 25.7 | 2.9 | — | — | 15 | 20 |
| Healthy | M | 71 | 18.5 | 3.8 | — | — | 18 | 25 |
| Healthy | F | 45 | 24.1 | 4.1 | — | — | 23 | 22 |
| Healthy | M | 50 | 22.3 | 9.2 | — | — | 32 | 30 |
| Healthy | F | 69 | 23.4 | 6.3 | — | — | 31 | 28 |
| Healthy | M | 63 | 24.7 | 5.1 | — | — | 35 | 21 |
| Healthy | M | 55 | 23.1 | 4.2 | — | — | 39 | 26 |
| Healthy | M | 54 | 22.6 | 2.7 | — | — | 18 | 19 |
| Healthy | M | 47 | 24.7 | 2.1 | — | — | 15 | 35 |
| Healthy | F | 43 | 23.6 | 2.3 | — | — | 24 | 28 |
| Healthy | F | 60 | 24.1 | 2.6 | — | — | 26 | 24 |
| Healthy | M | 51 | 19.6 | 2.9 | — | — | 22 | 25 |
| Healthy | M | 63 | 23.7 | 3.5 | — | — | 18 | 20 |
| Healthy | M | 66 | 25.8 | 4.3 | — | — | 13 | 19 |
| Healthy | M | 50 | 23.6 | 4.8 | — | — | 12 | 20 |
| Healthy | F | 58 | 23.1 | 5.2 | — | — | 21 | 24 |
| Healthy | M | 52 | 24.3 | 2.8 | — | — | 24 | 27 |
| Hepatitis | M | 58 | 24.2 | 5.2 | — | — | 25 | 18 |
| Hepatitis | F | 46 | 23.9 | 2.1 | — | — | 35 | 25 |
| Hepatitis | F | 53 | 22.4 | 2.0 | — | — | 29 | 32 |
| Hepatitis | F | 61 | 24.3 | 1.9 | — | — | 41 | 41 |
| Hepatitis | M | 49 | 22.8 | 3.8 | — | — | 18 | 26 |
| Hepatitis | F | 56 | 19.6 | 1.7 | — | — | 25 | 18 |
| Hepatitis | M | 65 | 20.3 | 2.4 | — | — | 29 | 19 |
| Hepatitis | F | 62 | 19.9 | 2.9 | — | — | 32 | 25 |
| Hepatitis | M | 71 | 23.6 | 3.6 | — | — | 35 | 27 |
| Hepatitis | F | 63 | 23.8 | 4.3 | — | — | 58 | 23 |
| Hepatitis | M | 52 | 22.7 | 4.5 | — | — | 32 | 36 |
| Hepatitis | F | 69 | 21.5 | 4.9 | — | — | 18 | 38 |
| Hepatitis | M | 54 | 23.6 | 5.3 | — | — | 24 | 34 |
| Hepatitis | F | 66 | 21.5 | 5.1 | — | — | 26 | 21 |
| Hepatitis | M | 57 | 19.6 | 2.7 | — | — | 27 | 27 |
| Hepatitis | M | 41 | 20.8 | 2.4 | — | — | 20 | 18 |
| Hepatitis | M | 60 | 22.7 | 3.5 | — | — | 18 | 46 |
| Hepatitis | M | 55 | 23.6 | 2.1 | — | — | 15 | 32 |
| Hepatitis | M | 50 | 26.1 | 2.0 | — | — | 26 | 18 |
| Hepatitis | F | 69 | 23.1 | 1.9 | — | — | 29 | 24 |
| Hepatitis | M | 61 | 23.9 | 2.8 | — | — | 38 | 22 |
| Hepatitis | M | 52 | 23.4 | 3.2 | — | — | 21 | 23 |
| Hepatitis | M | 59 | 22.7 | 2.4 | — | — | 32 | 20 |
| Hepatitis | M | 51 | 21.8 | 3.5 | — | — | 37 | 27 |
| Cirrhosis | F | 61 | 19.8 | 32.0 | — | A | 26 | 22 |
| Cirrhosis | F | 42 | 21.3 | 5.9 | — | A | 68 | 18 |
| Cirrhosis | F | 56 | 24.1 | 2.3 | — | A | 31 | 16 |
| Cirrhosis | F | 52 | 22.6 | 6.1 | — | A | 58 | 35 |
| Cirrhosis | F | 65 | 23.5 | 2.7 | — | A | 24 | 46 |
| Cirrhosis | F | 62 | 21.7 | 24.0 | — | A | 23 | 48 |
| Cirrhosis | M | 70 | 23.6 | 52.0 | — | A | 19 | 52 |
| Cirrhosis | M | 53 | 23.4 | 3.6 | — | A | 25 | 33 |
| Cirrhosis | F | 63 | 24.1 | 4.3 | — | A | 57 | 31 |
| Cirrhosis | M | 52 | 26.2 | 5.8 | — | A | 32 | 27 |
| Cirrhosis | M | 49 | 25.7 | 1.2 | — | A | 41 | 56 |
| Cirrhosis | M | 52 | 24.3 | 3.9 | — | A | 36 | 27 |
| Cirrhosis | F | 61 | 22.4 | 2.7 | — | A | 38 | 73 |
| Cirrhosis | M | 57 | 24.9 | 5.3 | — | A | 21 | 25 |
| Cirrhosis | M | 52 | 21.8 | 56.0 | — | A | 18 | 27 |
| Cirrhosis | M | 65 | 20.4 | 9.3 | — | A | 20 | 22 |
| Cirrhosis | M | 68 | 20.7 | 2.8 | — | A | 34 | 20 |
| Cirrhosis | M | 55 | 22.7 | 1.7 | — | A | 38 | 19 |
| Cirrhosis | M | 53 | 22.3 | 29.0 | — | A | 36 | 26 |
| Cirrhosis | M | 57 | 23.5 | 2.9 | — | A | 42 | 34 |
| Cirrhosis | M | 64 | 23.6 | 37.0 | — | A | 31 | 35 |
| Cirrhosis | M | 59 | 22.9 | 3.8 | — | A | 52 | 26 |
| Cirrhosis | M | 57 | 21.5 | 4.6 | — | A | 24 | 43 |
| Cirrhosis | M | 69 | 22.7 | 3.2 | — | A | 27 | 52 |
| HCC | M | 58 | 22.9 | 3260.0 | 3 | B | 33 | 32 |
| HCC | F | 65 | 21.8 | 20141.0 | 6.8 | B | 10 | 16 |
| HCC | M | 58 | 23.6 | 5249.0 | 8.6 | A | 43 | 42 |
| HCC | M | 51 | 24.5 | 30401.0 | 5 | A | 40 | 40 |
| HCC | M | 60 | 23.7 | 3.0 | 1 | A | 151 | 263 |
| HCC | M | 51 | 22.4 | 137.9 | 6 | A | 48 | 38 |
| HCC | M | 51 | 26.3 | 1065.0 | 7 | A | 89 | 60 |
| HCC | F | 59 | 21.8 | 28088.0 | 15 | A | 63 | 52 |
| HCC | M | 66 | 19.6 | 704.5 | 4.8 | A | 24 | 37 |
| HCC | M | 57 | 18.4 | 3278.0 | 10.7 | A | 28 | 41 |
| HCC | M | 51 | 23.5 | 3.6 | 4.2 | B | 18 | 36 |
| HCC | M | 43 | 24.1 | 58478.0 | 12.6 | A | 16 | 28 |
| HCC | M | 52 | 24.7 | 122.4 | 5 | A | 25 | 20 |
| HCC | M | 48 | 23.1 | 790.8 | 7 | A | 37 | 18 |
| HCC | M | 46 | 20.5 | 42593.0 | 6 | B | 201 | 105 |
| HCC | M | 56 | 22.6 | 8.5 | 6 | A | 51 | 63 |
| HCC | M | 54 | 23.4 | 653.0 | 3.2 | A | 27 | 35 |
| HCC | M | 44 | 24.8 | 595.8 | 2.4 | A | 23 | 28 |
| HCC | M | 59 | 22.7 | 55710.0 | 10 | B | 56 | 80 |
| HCC | M | 54 | 22.5 | 690.3 | 2.4 | A | 52 | 16 |
| HCC | M | 44 | 21.4 | 18.4 | 10 | A | 27 | 22 |
| HCC | M | 61 | 19.7 | 219.0 | 5 | A | 58 | 28 |
| HCC | M | 44 | 19.5 | 69.0 | 1.8 | A | 42 | 35 |
| HCC | M | 29 | 20.4 | 3486.0 | 11 | A | 51 | 57 |
| HCC | M | 52 | 20.3 | 168.1 | 1 | B | 18 | 35 |
| HCC | M | 42 | 26.1 | 18267.0 | 9 | A | 23 | 23 |
| HCC | M | 62 | 21.7 | 11.3 | 6 | A | 27 | 28 |
| HCC | M | 63 | 21.6 | 8.3 | 11 | A | 57 | 69 |
| HCC | F | 59 | 22.4 | 1698.0 | 3 | A | 29 | 36 |
| HCC | F | 52 | 22.6 | 209.1 | 12 | A | 38 | 48 |
| HCC | F | 45 | 23.6 | 10601.0 | 8 | A | 76 | 152 |
| HCC | F | 63 | 22.5 | 1.8 | 5 | A | 72 | 64 |
| HCC | F | 64 | 23.6 | 4.8 | 6 | B | 34 | 18 |
| HCC | F | 68 | 23.7 | 5.4 | 6 | A | 39 | 21 |
| HCC | F | 66 | 23.1 | 2766.0 | 8 | A | 28 | 27 |
| HCC | M | 58 | 23.5 | 17.2 | 1.3 | A | 22 | 23 |
| HCC | M | 67 | 23.8 | 37.8 | 3 | A | 19 | 52 |
| HCC | M | 59 | 21.6 | 436.9 | 3 | B | 36 | 26 |
| HCC | M | 53 | 24.3 | 28.5 | 2 | A | 24 | 89 |
| HCC | M | 69 | 25.2 | 4.7 | 4 | A | 67 | 74 |
| HCC | M | 54 | 24.7 | 239.2 | 7 | B | 52 | 56 |
| HCC | M | 70 | 21.6 | 2.9 | 6.7 | B | 28 | 21 |
| HCC | M | 57 | 21.9 | 2023.0 | 4 | A | 28 | 28 |
| HCC | M | 73 | 20.9 | 6.1 | 3 | A | 15 | 16 |
| HCC | M | 58 | 20.7 | 3447.0 | 7 | A | 17 | 38 |
| HCC | M | 57 | 21.6 | 9.0 | 10 | A | 26 | 84 |
| HCC | F | 62 | 22.5 | 45.1 | 1.2 | A | 29 | 26 |
| HCC | F | 63 | 23.4 | 27268.0 | 11 | A | 36 | 49 |
| HCC | F | 63 | 26.1 | 172.1 | 3 | B | 37 | 57 |
| HCC | F | 64 | 21.5 | 1436.0 | 7 | A | 35 | 63 |
| HCC | F | 64 | 19.8 | 165.8 | 2 | A | 318 | 117 |
| HCC | F | 64 | 21.2 | 777.0 | 8 | A | 130 | 106 |
| HCC | F | 69 | 18.3 | 592.5 | 3 | A | 34 | 25 |
| HCC | F | 68 | 22.7 | 3.1 | 5 | A | 52 | 28 |
| HCC | F | 59 | 20.8 | 2701.0 | 7 | B | 58 | 23 |
| HCC | F | 68 | 22.4 | 289.6 | 4 | A | 35 | 17 |
| HCC | F | 67 | 22.1 | 63.8 | 4 | A | 17 | 36 |
| HCC | M | 53 | 22.6 | 33.4 | 3 | A | 24 | 38 |
| HCC | F | 64 | 22.8 | 5.2 | 2 | A | 29 | 18 |
| HCC | M | 52 | 23.5 | 2487.0 | 5 | A | 26 | 20 |
| HCC | M | 54 | 23.4 | 29.8 | 4.3 | A | 39 | 34 |
| HCC | M | 53 | 22.3 | 5.8 | 5.7 | A | 42 | 45 |
| HCC | M | 58 | 23.2 | 589.1 | 12.2 | A | 26 | 49 |
| HCC | M | 80 | 23.7 | 16.4 | 6 | A | 35 | 23 |
| HCC | M | 70 | 21.6 | 4.1 | 3 | B | 28 | 36 |
| HCC | M | 68 | 25.3 | 97.2 | 12 | B | 39 | 38 |
| HCC | F | 57 | 21.7 | 3.7 | 13 | A | 43 | 96 |
| HCC | F | 66 | 22.6 | 7886.0 | 8 | A | 57 | 55 |
| HCC | F | 72 | 18.2 | 77.0 | 2 | A | 114 | 143 |
| HCC | M | 56 | 21.3 | 910.0 | 2.2 | A | 21 | 63 |
| HCC | M | 54 | 24.9 | 126.8 | 10 | A | 34 | 25 |
| HCC | M | 61 | 25.3 | 4.1 | 6 | B | 23 | 23 |
| HCC | M | 66 | 23.4 | 74.5 | 5 | A | 28 | 17 |
| HCC | M | 48 | 21.4 | 156.2 | 1.1 | A | 16 | 42 |
| HCC | M | 70 | 23.1 | 39.3 | 8 | A | 29 | 59 |

| **GGT**  **(10-60 U/L)** | **Total protein**  **(65-85 g/L)** | **Albumin**  **(40-55 g/L)** | **Globulin**  **(20-40 g/L)** | **Total bilirubin**  **(6.8-30 umol/L)** | **Direct bilirubin**  **(0-8.6 umol/L)** | **Cirrhosis**  **(Y or N)** | **Cause of disease** | **Dietary habit** |
| --- | --- | --- | --- | --- | --- | --- | --- | --- |
| 20 | 70.2 | 50.7 | 31.8 | 17.9 | 5.9 | — | — | mixed diet |
| 28 | 73.4 | 52.1 | 32.4 | 14.6 | 4.8 | — | — | mixed diet |
| 31 | 78.1 | 49.6 | 26.8 | 15.4 | 4.7 | — | — | mixed diet |
| 23 | 69.8 | 43.5 | 24.7 | 13.2 | 4.4 | — | — | mixed diet |
| 28 | 72.5 | 44.8 | 25.6 | 12.5 | 3.1 | — | — | mixed diet |
| 26 | 70.1 | 49.3 | 23.9 | 13.9 | 4.3 | — | — | mixed diet |
| 33 | 71.4 | 51.4 | 30.2 | 15.3 | 4.5 | — | — | mixed diet |
| 26 | 70.6 | 50.3 | 25.1 | 19.6 | 4.8 | — | — | mixed diet |
| 15 | 72.4 | 49.8 | 32.1 | 23.6 | 8.9 | — | — | mixed diet |
| 21 | 75.3 | 46.9 | 20.4 | 18.2 | 5.7 | — | — | mixed diet |
| 17 | 71 | 48.4 | 23.1 | 12.1 | 4.2 | — | — | mixed diet |
| 25 | 69.4 | 48.2 | 22.5 | 14.8 | 4.3 | — | — | mixed diet |
| 24 | 70.7 | 49.6 | 24.9 | 15.7 | 6.4 | — | — | mixed diet |
| 20 | 73.5 | 53.1 | 30.5 | 17.6 | 4.8 | — | — | mixed diet |
| 26 | 74.1 | 52.7 | 27.1 | 16.8 | 5 | — | — | mixed diet |
| 15 | 74.5 | 50.2 | 26.1 | 13.6 | 3.9 | — | — | mixed diet |
| 16 | 73.2 | 47.6 | 23.5 | 16.3 | 5.1 | — | — | mixed diet |
| 18 | 78.9 | 45.3 | 20.2 | 15.9 | 4.1 | — | — | mixed diet |
| 21 | 74.1 | 48.8 | 23.8 | 18.4 | 5.2 | — | — | mixed diet |
| 23 | 75.6 | 52.8 | 25.9 | 16.5 | 4.1 | — | — | mixed diet |
| 26 | 69.8 | 48.7 | 30.5 | 12.8 | 4.2 | — | HBV | mixed diet |
| 31 | 73.6 | 53.2 | 27.9 | 23.6 | 8.1 | — | HBV | mixed diet |
| 38 | 79.4 | 51.1 | 32.4 | 21.3 | 7.2 | — | HBV | mixed diet |
| 45 | 75.9 | 49 | 26.3 | 19.4 | 5.7 | — | HBV | mixed diet |
| 22 | 70.3 | 50.9 | 27.8 | 13.2 | 3.8 | — | HBV | mixed diet |
| 20 | 72.1 | 46.8 | 20.3 | 15.4 | 4.2 | — | HBV | mixed diet |
| 24 | 70.6 | 50.7 | 26.4 | 18.2 | 5.7 | — | HBV | mixed diet |
| 21 | 74.5 | 52.1 | 21.6 | 21.3 | 5.9 | — | HBV | mixed diet |
| 32 | 76.1 | 45.3 | 23.3 | 16.4 | 4.4 | — | HCV | mixed diet |
| 33 | 70.2 | 38.8 | 20.1 | 20.4 | 5.9 | — | HCV | mixed diet |
| 41 | 73.4 | 55.4 | 30.2 | 15.8 | 4.8 | — | HCV | mixed diet |
| 24 | 69.9 | 52.3 | 25.3 | 16.9 | 5.2 | — | HCV | mixed diet |
| 20 | 71.8 | 43 | 26.1 | 22 | 6.5 | — | HCV | mixed diet |
| 26 | 76.3 | 41 | 27.9 | 10.8 | 3.1 | — | HCV | mixed diet |
| 29 | 70.2 | 50.9 | 29.1 | 20.2 | 5.8 | — | HCV | mixed diet |
| 32 | 71.9 | 49.7 | 24.3 | 22.1 | 6.2 | — | HCV | mixed diet |
| 45 | 78.4 | 24.5 | 22.3 | 19.7 | 5.6 | — | Alcohol | mixed diet |
| 44 | 72.2 | 30.2 | 20.4 | 14.3 | 4.5 | — | Alcohol | mixed diet |
| 36 | 68.4 | 35.4 | 21.8 | 13.3 | 4.2 | — | Alcohol | mixed diet |
| 31 | 71.1 | 50.1 | 30.2 | 28.8 | 8.6 | — | Alcohol | mixed diet |
| 29 | 75.1 | 49.6 | 27.8 | 18.4 | 5.7 | — | Alcohol | mixed diet |
| 27 | 73.6 | 48.7 | 24.6 | 13.5 | 4.2 | — | Alcohol | mixed diet |
| 36 | 77.2 | 47.3 | 23.5 | 16.4 | 5.1 | — | Alcohol | mixed diet |
| 48 | 70.4 | 48.1 | 25.5 | 15.5 | 4.9 | — | Alcohol | mixed diet |
| 37 | 70.6 | 43.2 | 31.3 | 10.9 | 8.8 | Y | HBV | mixed diet |
| 29 | 74.1 | 53.6 | 34.2 | 19.5 | 12.1 | Y | HBV | mixed diet |
| 59 | 68.9 | 49.1 | 30.5 | 27.9 | 4.9 | Y | HBV | mixed diet |
| 62 | 72.5 | 39.2 | 26.4 | 10.5 | 9.6 | Y | HBV | mixed diet |
| 52 | 62.4 | 50.7 | 30.3 | 23.4 | 5.5 | Y | HBV | mixed diet |
| 42 | 58.6 | 46.5 | 24.6 | 12 | 7.2 | Y | HBV | mixed diet |
| 48 | 75.4 | 41.3 | 35.1 | 18.5 | 6.9 | Y | HBV | mixed diet |
| 47 | 71.3 | 46.9 | 25.9 | 17.3 | 8.5 | Y | HBV | mixed diet |
| 59 | 72.4 | 33.3 | 31 | 21.7 | 7.4 | Y | HCV | mixed diet |
| 68 | 69.1 | 53.6 | 31.7 | 17.9 | 11 | Y | HCV | mixed diet |
| 52 | 74.3 | 48.6 | 22.5 | 26.4 | 11.1 | Y | HCV | mixed diet |
| 47 | 70.2 | 37.3 | 33.6 | 28.2 | 6.9 | Y | HCV | mixed diet |
| 82 | 75.1 | 51 | 28.9 | 15.4 | 11.5 | Y | HCV | mixed diet |
| 30 | 71.9 | 41.6 | 32.5 | 28.2 | 9.4 | Y | HCV | mixed diet |
| 25 | 72.3 | 47.1 | 30.4 | 24.1 | 13.9 | Y | HCV | mixed diet |
| 36 | 67.5 | 34.9 | 32.8 | 30.1 | 8.9 | Y | HCV | mixed diet |
| 41 | 78.8 | 45.1 | 24.7 | 22.1 | 7.8 | Y | Alcohol | mixed diet |
| 35 | 79.6 | 52.7 | 30.6 | 18.5 | 10.1 | Y | Alcohol | mixed diet |
| 39 | 77.1 | 32.9 | 29.3 | 24.9 | 7.5 | Y | Alcohol | mixed diet |
| 59 | 72.4 | 35.2 | 30.7 | 19.6 | 13.2 | Y | Alcohol | mixed diet |
| 63 | 70.8 | 42.1 | 30 | 30.2 | 9.9 | Y | Alcohol | mixed diet |
| 68 | 73.6 | 45.5 | 27.6 | 23.3 | 7.4 | Y | Alcohol | mixed diet |
| 34 | 74.6 | 40.9 | 32.8 | 18.8 | 7.5 | Y | Alcohol | mixed diet |
| 78 | 75.3 | 38.4 | 25.9 | 18.7 | 6.2 | Y | Alcohol | mixed diet |
| 63 | 74 | 32.5 | 28.6 | 42 | 10.3 | Y | HBV | mixed diet |
| 20 | 58.2 | 40.7 | 30.3 | 68.7 | 19.6 | Y | HBV | mixed diet |
| 65 | 70.6 | 34.2 | 24.3 | 32.1 | 6.3 | Y | HBV | mixed diet |
| 62 | 69.3 | 28 | 22 | 9.4 | 2.3 | Y | HBV | mixed diet |
| 358 | 71.8 | 43 | 27.6 | 15.8 | 4.1 | N | HBV | mixed diet |
| 54 | 69.1 | 35.8 | 30.6 | 8 | 1.9 | N | HBV | mixed diet |
| 98 | 58.4 | 33.8 | 27.8 | 4.1 | 0.6 | Y | HBV | mixed diet |
| 84 | 62.3 | 36.1 | 26.9 | 19.4 | 4.6 | Y | HBV | mixed diet |
| 59 | 77.1 | 32.4 | 30.2 | 9.4 | 2.2 | N | HBV | mixed diet |
| 63 | 70.6 | 42.2 | 30.3 | 39.7 | 10.8 | Y | HBV | mixed diet |
| 52 | 70.2 | 36 | 28.4 | 61.9 | 18.6 | Y | HBV | mixed diet |
| 34 | 72.4 | 44 | 29.6 | 19.4 | 5.1 | Y | HBV | mixed diet |
| 28 | 65.3 | 47.5 | 31.2 | 15.42 | 3.7 | N | HBV | mixed diet |
| 26 | 68.1 | 41.7 | 25.7 | 29.3 | 6.3 | Y | HBV | mixed diet |
| 208 | 73.5 | 32.5 | 29.2 | 73.6 | 19.1 | Y | HBV | mixed diet |
| 82 | 70.2 | 43.3 | 27.9 | 14.4 | 3.2 | N | HBV | mixed diet |
| 48 | 58.5 | 39 | 26.3 | 27 | 5.9 | Y | HBV | mixed diet |
| 36 | 71.4 | 34.8 | 32.5 | 9.9 | 2.5 | Y | HBV | mixed diet |
| 96 | 58.9 | 31.2 | 29.3 | 74.4 | 20.1 | Y | HBV | mixed diet |
| 72 | 63.5 | 40.5 | 31.3 | 10 | 2.2 | Y | HBV | mixed diet |
| 35 | 76.4 | 31.2 | 29.1 | 26.3 | 7.9 | Y | HBV | mixed diet |
| 69 | 66.1 | 39.1 | 30.6 | 33.5 | 8.5 | Y | HBV | mixed diet |
| 38 | 70.8 | 40.3 | 32.8 | 17.4 | 4.5 | N | HBV | mixed diet |
| 29 | 71.4 | 36.8 | 29.8 | 21.4 | 5.6 | Y | HBV | mixed diet |
| 56 | 64 | 46.3 | 41.2 | 54.7 | 14.6 | Y | HBV | mixed diet |
| 31 | 63.9 | 38.3 | 32.3 | 29.2 | 8.9 | Y | HBV | mixed diet |
| 39 | 57.3 | 46 | 35.9 | 23.9 | 6.2 | N | HBV | mixed diet |
| 95 | 59.9 | 34.4 | 29.8 | 15.8 | 4.3 | Y | HBV | mixed diet |
| 32 | 74.2 | 32.6 | 27.6 | 17.2 | 4.8 | Y | HBV | mixed diet |
| 41 | 71.6 | 33 | 22.4 | 16 | 4.6 | N | HBV | mixed diet |
| 162 | 71 | 33.8 | 26.5 | 13.9 | 3.7 | Y | HBV | mixed diet |
| 75 | 56.6 | 32.5 | 29.1 | 29.9 | 8.8 | Y | HBV | mixed diet |
| 34 | 63.2 | 25.5 | 23.1 | 64.6 | 19.6 | Y | HBV | mixed diet |
| 45 | 68.9 | 32.6 | 27.6 | 20.2 | 5.9 | Y | HBV | mixed diet |
| 41 | 70.1 | 36.9 | 29.6 | 18.1 | 4.9 | Y | HBV | mixed diet |
| 39 | 74.2 | 45.2 | 31.8 | 14.5 | 3.8 | Y | HCV | mixed diet |
| 67 | 67.6 | 38 | 30 | 9.7 | 3 | Y | HCV | mixed diet |
| 41 | 59.3 | 27.9 | 25.1 | 59.3 | 16.3 | Y | HCV | mixed diet |
| 94 | 64.7 | 41.5 | 32.6 | 18.2 | 4.2 | N | HCV | mixed diet |
| 101 | 68.2 | 40.1 | 28.6 | 15.4 | 3.2 | N | HCV | mixed diet |
| 69 | 60.5 | 26.4 | 24.1 | 135.6 | 40.7 | Y | HCV | mixed diet |
| 38 | 74.5 | 39.1 | 32.5 | 56.5 | 15.1 | Y | HCV | mixed diet |
| 30 | 70.3 | 36.4 | 30.4 | 17.9 | 4.5 | N | HCV | mixed diet |
| 28 | 73.2 | 34.3 | 29.8 | 25.5 | 7.3 | N | HCV | mixed diet |
| 41 | 59.8 | 38.1 | 27.6 | 19.3 | 5.3 | Y | HCV | mixed diet |
| 79 | 65.3 | 40.9 | 30.4 | 25.9 | 7.6 | Y | HCV | mixed diet |
| 43 | 68.7 | 32.9 | 27.4 | 18 | 5.2 | N | HCV | mixed diet |
| 62 | 66.9 | 30.1 | 26.9 | 51 | 14.8 | Y | HCV | mixed diet |
| 68 | 73.6 | 32.7 | 25.5 | 34.3 | 9.6 | Y | HCV | mixed diet |
| 59 | 55.5 | 36.4 | 30.2 | 17 | 3.9 | N | HCV | mixed diet |
| 392 | 73.2 | 26.2 | 24.7 | 30.2 | 9.8 | Y | HCV | mixed diet |
| 152 | 58.2 | 34.6 | 30.5 | 31 | 10 | Y | HCV | mixed diet |
| 34 | 58.9 | 37.6 | 31.8 | 13.7 | 3.7 | N | HCV | mixed diet |
| 62 | 67.3 | 41.6 | 33.9 | 11.9 | 2.9 | N | HCV | mixed diet |
| 69 | 76.2 | 24 | 20.2 | 56.1 | 18.2 | Y | HCV | mixed diet |
| 47 | 59.1 | 29.2 | 26.6 | 18 | 5.1 | Y | HCV | mixed diet |
| 53 | 72.3 | 27.9 | 25.4 | 27.2 | 5.9 | Y | HCV | mixed diet |
| 39 | 60.1 | 40.1 | 32.6 | 16.3 | 4.1 | Y | HCV | mixed diet |
| 26 | 65.3 | 27.8 | 22.8 | 27.6 | 5.5 | Y | HCV | mixed diet |
| 18 | 66.8 | 38.1 | 31.5 | 29.8 | 10.4 | Y | HCV | mixed diet |
| 47 | 73.9 | 27.3 | 24.9 | 15.4 | 4.2 | N | Alcohol | mixed diet |
| 62 | 59.3 | 37.3 | 28.9 | 25.5 | 5.1 | Y | Alcohol | mixed diet |
| 53 | 64.7 | 30.6 | 26.7 | 27.4 | 5.9 | N | Alcohol | mixed diet |
| 28 | 68.6 | 29.2 | 25.9 | 10.3 | 2.5 | N | Alcohol | mixed diet |
| 42 | 72.4 | 39.1 | 30.6 | 42.7 | 11.5 | N | Alcohol | mixed diet |
| 71 | 74.3 | 32.2 | 29.2 | 65.2 | 19.7 | N | Alcohol | mixed diet |
| 103 | 63.4 | 39.9 | 33.6 | 12.5 | 3.5 | N | Alcohol | mixed diet |
| 75 | 57.2 | 40.2 | 36.9 | 20.8 | 6.9 | Y | Alcohol | mixed diet |
| 186 | 59 | 35.7 | 32.1 | 9.2 | 2.1 | Y | Alcohol | mixed diet |
| 39 | 77.2 | 47 | 38.8 | 12.2 | 3.5 | Y | Alcohol | mixed diet |
| 47 | 63.3 | 34 | 30.2 | 9.1 | 2.2 | Y | Alcohol | mixed diet |
| 39 | 64.2 | 22.6 | 20.6 | 48.5 | 14.7 | Y | Alcohol | mixed diet |
| 49 | 70.8 | 41 | 34.2 | 11.15 | 3.2 | Y | Alcohol | mixed diet |
| 50 | 61.4 | 37.2 | 30.6 | 26.2 | 6.9 | Y | Alcohol | mixed diet |
| 69 | 69.3 | 43.8 | 38.1 | 27.4 | 6.3 | N | Alcohol | mixed diet |

**Table S2. Different degree of genera levels in Healthy, Hepatitis, Cirrhosis and HCC**

| **Feature** | **P values** | **FDR** | **GA** | **YH** | **YZ** | **ZC** | **LDA score** |
| --- | --- | --- | --- | --- | --- | --- | --- |
| Phyllobacterium | 2.60E-15 | 2.05E-13 | 22032 | 98794 | 69152 | 812.07 | 4.69 |
| Sphingomonas | 8.53E-13 | 3.37E-11 | 3026.1 | 12162 | 6313.7 | 267.29 | 3.77 |
| Enterococcus | 2.18E-10 | 5.75E-09 | 42818 | 457970 | 25660 | 13241 | 5.35 |
| Neisseria | 8.07E-09 | 1.29E-07 | 171.47 | 9236.5 | 20330 | 8759.9 | 4 |
| Lachnospira | 8.19E-09 | 1.29E-07 | 45751 | 25893 | 51403 | 87183 | 4.49 |
| Ralstonia | 1.64E-07 | 2.16E-06 | 1398.8 | 3561.6 | 2284.9 | 620.49 | 3.17 |
| Bradyrhizobium | 7.1211E-7 | 5.6424E-6 | 405.94 | 793.83 | 532.23 | 25.187 | 2.59 |
| Erysipelatoclostridium | 6.47E-07 | 5.73E-06 | 7524.4 | 20086 | 2204.8 | 3617 | 3.95 |
| Sarcina | 6.85E-07 | 5.73E-06 | 2766.6 | 1796.6 | 180.58 | 652.6 | 3.11 |
| Catenibacterium | 7.24E-07 | 5.73E-06 | 10592 | 851.23 | 8679.6 | 39941 | 4.29 |
| Marvinbryantia | 7.34E-07 | 5.73E-06 | 2753.4 | 858.26 | 1253.6 | 3050.9 | 3.04 |
| Mitsuokella | 7.98E-07 | 5.73E-06 | 5034.5 | 6379.3 | 100280 | 19162 | 4.68 |
| Romboutsia | 1.18E-06 | 7.78E-06 | 38639 | 18666 | 39101 | 54902 | 4.26 |
| Allisonella | 2.13E-06 | 1.20E-05 | 1874.9 | 1026 | 1780 | 4207.1 | 3.2 |
| Butyricicoccus | 2.13E-06 | 1.20E-05 | 19645 | 21278 | 40231 | 26360 | 4.01 |
| Caproiciproducens | 2.89E-06 | 1.52E-05 | 5716.6 | 416.38 | 2071.1 | 3803.5 | 3.42 |
| Hungatella | 3.43E-06 | 1.70E-05 | 9529.5 | 16607 | 5156.4 | 3825.4 | 3.81 |
| Megamonas | 3.88E-06 | 1.80E-05 | 122900 | 288940 | 758350 | 76959 | 5.53 |
| Oscillibacter | 4.21E-06 | 1.85E-05 | 6031.7 | 5375.7 | 12378 | 4370.7 | 3.6 |
| Negativibacillus | 6.04E-06 | 2.51E-05 | 487.74 | 2543.5 | 1817.2 | 1371.4 | 3.01 |
| Coprobacter | 1.21E-05 | 4.77E-05 | 2181.7 | 236.54 | 427.19 | 191.88 | 3 |
| Adlercreutzia | 2.31E-05 | 8.39E-05 | 3593.4 | 1665 | 1737 | 3908.3 | 3.05 |
| Paraprevotella | 2.34E-05 | 8.39E-05 | 50268 | 12198 | 24936 | 13958 | 4.28 |
| Fusicatenibacter | 3.36E-05 | 0.000116 | 91770 | 40945 | 95726 | 108590 | 4.53 |
| Intestinibacter | 4.75E-05 | 0.000156 | 39890 | 35000 | 28129 | 45602 | 3.94 |
| Blautia | 5.19E-05 | 0.000164 | 490100 | 217170 | 362990 | 429600 | 5.14 |
| Bilophila | 5.71E-05 | 0.000174 | 9386.1 | 4213.5 | 7082 | 9294.3 | 3.41 |
| Megasphaera | 7.94E-05 | 0.000232 | 35352 | 29968 | 133830 | 4896.9 | 4.81 |
| Weissella | 9.67E-05 | 0.000273 | 4337.3 | 3972.9 | 5229.6 | 10119 | 3.49 |
| Tyzzerella | 0.00011909 | 0.000324 | 15251 | 12990 | 7889.9 | 12799 | 3.57 |
| Akkermansia | 0.00013397 | 0.000353 | 19054 | 234770 | 16443 | 3290.1 | 5.06 |
| Dialister | 0.00015917 | 0.000406 | 81116 | 122180 | 144230 | 367730 | 5.16 |
| Delftia | 0.00022407 | 0.000553 | 3808.3 | 9088.5 | 4529.4 | 2421.1 | 3.52 |
| Bifidobacterium | 0.00040644 | 0.000966 | 127570 | 75419 | 155030 | 117020 | 4.6 |
| Dorea | 0.00041592 | 0.000966 | 48585 | 37202 | 61595 | 62654 | 4.1 |
| Intestinimonas | 0.0008203 | 0.001852 | 9199.1 | 4123.2 | 7786.5 | 9797.5 | 3.45 |
| Sutterella | 0.0015514 | 0.003405 | 10652 | 7905.7 | 5982.7 | 27467 | 4.03 |
| Turicibacter | 0.0019033 | 0.004064 | 16811 | 6958.5 | 7917.2 | 13990 | 3.69 |
| Peptostreptococcus | 0.0020371 | 0.004235 | 376.7 | 789.73 | 705.91 | 401.41 | 2.32 |
| Faecalibacterium | 0.0026277 | 0.005323 | 1186000 | 836210 | 1404900 | 1740700 | 5.66 |
| Flavonifractor | 0.0037243 | 0.007355 | 16074 | 18541 | 10463 | 9115.1 | 3.67 |
| Enterobacteriaceae | 0.0047666 | 0.009184 | 580100 | 691980 | 125110 | 205640 | 5.45 |
| Acidaminococcus | 0.0057572 | 0.010829 | 2849.8 | 2551.3 | 9628.7 | 59367 | 4.45 |
| Eisenbergiella | 0.0068447 | 0.012575 | 2787.9 | 879.28 | 477.33 | 1754.6 | 3.06 |
| Collinsella | 0.0073836 | 0.013257 | 24352 | 70893 | 50862 | 53285 | 4.37 |
| Clostridiales | 0.0080009 | 0.014046 | 97538 | 38145 | 70766 | 68443 | 4.47 |
| Phascolarctobacterium | 0.008199 | 0.014081 | 179970 | 111080 | 94456 | 172350 | 4.63 |
| Erysipelotrichaceae | 0.013248 | 0.021805 | 42775 | 28543 | 37336 | 92759 | 4.51 |
| Veillonella | 0.014082 | 0.022704 | 99948 | 237020 | 46640 | 32607 | 5.01 |
| Holdemanella | 0.026626 | 0.042069 | 48898 | 25369 | 100800 | 21977 | 4.6 |
| Sellimonas | 0.029375 | 0.045502 | 4737.2 | 1213.1 | 2297.4 | 5001.3 | 3.28 |
| Desulfovibrio | 0.042989 | 0.065311 | 13942 | 5368.5 | 10532 | 7118.5 | 3.63 |
| Prevotellaceae | 0.043854 | 0.065367 | 1141100 | 516740 | 914190 | 1201800 | 5.53 |
| Parasutterella | 0.052912 | 0.077409 | 49210 | 27101 | 32693 | 60112 | 4.22 |
| Faecalitalea | 0.061208 | 0.087917 | 2414.8 | 957.55 | 695.7 | 1389.8 | 2.93 |
| Gemella | 0.067133 | 0.094706 | 1111.1 | 2300.9 | 2775 | 1293.6 | 2.92 |
| Senegalimassilia | 0.070689 | 0.09631 | 1418.9 | 803.5 | 1464.8 | 2616 | 2.96 |
| Odoribacter | 0.071215 | 0.09631 | 2925.6 | 3702.5 | 2472.2 | 4885.2 | 3.08 |
| Ruminococcaceae | 0.071928 | 0.09631 | 263110 | 194000 | 276730 | 270560 | 4.62 |
| Alistipes | 0.073715 | 0.097058 | 63796 | 40667 | 50464 | 33570 | 4.18 |
| Bacteroides | 0.11833 | 0.15325 | 1886400 | 2536500 | 1972500 | 1560700 | 5.69 |
| Eggerthella | 0.16011 | 0.20401 | 11211 | 2618 | 1536.6 | 3318.9 | 3.68 |
| Butyricimonas | 0.16435 | 0.20609 | 1583.3 | 2815.5 | 2730.3 | 1421.8 | 2.84 |
| Roseburia | 0.18787 | 0.2319 | 198890 | 159100 | 217410 | 214750 | 4.46 |
| Not_Assigned | 0.20489 | 0.24902 | 1398600 | 1177000 | 1213400 | 1312200 | 5.04 |
| Actinomyces | 0.28369 | 0.33956 | 1808.1 | 1820.8 | 2596.4 | 1314.2 | 2.81 |
| Lactobacillus | 0.30169 | 0.35573 | 85479 | 112060 | 70862 | 74653 | 4.31 |
| Fusobacterium | 0.36346 | 0.42225 | 49641 | 16706 | 6721.2 | 9755.2 | 4.33 |
| Alloprevotella | 0.3875 | 0.44366 | 80566 | 29268 | 78089 | 59296 | 4.41 |
| Lachnospiraceae | 0.42336 | 0.4778 | 343390 | 408550 | 362190 | 437000 | 4.67 |
| Lachnoclostridium | 0.45465 | 0.50587 | 140260 | 128340 | 138530 | 155440 | 4.13 |
| Anaerostipes | 0.55247 | 0.60619 | 52143 | 49784 | 46662 | 50065 | 3.44 |
| Agathobacter | 0.62604 | 0.67749 | 3885.5 | 2957.9 | 3345.6 | 4599.9 | 2.91 |
| Barnesiella | 0.67266 | 0.71811 | 12663 | 13612 | 8708.4 | 6937.4 | 3.52 |
| Subdoligranulum | 0.71005 | 0.74791 | 111230 | 106860 | 91984 | 103140 | 3.98 |
| Streptococcus | 0.86548 | 0.89964 | 217880 | 350610 | 154140 | 223430 | 4.99 |
| Coprobacillus | 0.90065 | 0.92405 | 7633.1 | 1750.3 | 1549.2 | 1549.6 | 3.48 |
| Parabacteroides | 0.95888 | 0.97118 | 144260 | 178590 | 142410 | 117100 | 4.49 |
| Terrisporobacter | 0.99129 | 0.99129 | 18509 | 15504 | 16537 | 19811 | 3.33 |

FDR, False Discovery Rate; HCC, Hepatocellular carcinoma; LDA, linear discriminant analysis.

**Table S3. Different degree of genera levels in Cirrhosis，HCC with Cirrhosis and HCC no Cirrhosis**

| **Feature** | **P values** | **FDR** | **HCC with Cirrhosis** | **HCC no Cirrhosis** | **Cirrhosis** | **LDA score** |
| --- | --- | --- | --- | --- | --- | --- |
| Enterococcus | 1.30E-07 | 1.04E-05 | 37630 | 54452 | 458170 | 5.32 |
| Marvinbryantia | 8.13E-06 | 0.00026 | 2801.8 | 2645.7 | 859 | 2.99 |
| Alphaproteobacteria | 9.74E-06 | 0.00026 | 1553.1 | 2905.2 | 0 | 3.16 |
| Phyllobacterium | 1.32E-05 | 0.000263 | 22051 | 22153 | 98794 | 4.58 |
| Sphingomonas | 4.94E-05 | 0.00079 | 3161.9 | 2749 | 12162 | 3.67 |
| Blautia | 9.81E-05 | 0.001308 | 543710 | 369600 | 217410 | 5.21 |
| Adlercreutzia | 0.000206 | 0.002066 | 4089.8 | 2472.6 | 1665.4 | 3.08 |
| Intestinibacter | 0.000207 | 0.002066 | 37993 | 44347 | 35066 | 3.67 |
| Bilophila | 0.000263 | 0.002226 | 9007.3 | 10261 | 4215.3 | 3.48 |
| Romboutsia | 0.000297 | 0.002226 | 35319 | 46465 | 18685 | 4.14 |
| Intestinimonas | 0.000306 | 0.002226 | 7942.5 | 12081 | 4127.1 | 3.6 |
| Lachnospira | 0.00049 | 0.003267 | 45449 | 46691 | 25917 | 4.02 |
| Peptococcus | 0.000671 | 0.003896 | 400.67 | 651.64 | 124.28 | 2.42 |
| Akkermansia | 0.000682 | 0.003896 | 15490 | 27132 | 234810 | 5.04 |
| Negativibacillus | 0.00105 | 0.005411 | 381.33 | 733.02 | 2547.2 | 3.04 |
| Peptostreptococcus | 0.001082 | 0.005411 | 414 | 293.94 | 790.6 | 2.4 |
| Erysipelatoclostridium | 0.001607 | 0.007563 | 9804.7 | 2380 | 20084 | 3.95 |
| Coprobacter | 0.002079 | 0.009239 | 2128.3 | 2339.3 | 236.55 | 3.02 |
| Turicibacter | 0.003596 | 0.015143 | 14194 | 22891 | 6968.9 | 3.9 |
| Paraprevotella | 0.004944 | 0.019649 | 32985 | 89338 | 12227 | 4.59 |
| Ralstonia | 0.005158 | 0.019649 | 1507.6 | 1158.1 | 3563 | 3.08 |
| Fusicatenibacter | 0.005849 | 0.021268 | 101100 | 70715 | 40970 | 4.48 |
| unidentified_Clostridiales | 0.007907 | 0.027504 | 105670 | 86104 | 38189 | 4.53 |
| Delftia | 0.009453 | 0.031285 | 3766.8 | 3948.5 | 9090 | 3.43 |
| Papillibacter | 0.009777 | 0.031285 | 553.88 | 1657.8 | 146.97 | 2.88 |
| Phascolarctobacterium | 0.011784 | 0.036259 | 174060 | 193740 | 111190 | 4.62 |
| unidentified_Prevotellaceae | 0.019234 | 0.05699 | 1137800 | 1181200 | 518860 | 5.52 |
| Rothia | 0.024694 | 0.070554 | 888.42 | 984.58 | 2178.4 | 2.81 |
| Gemella | 0.029114 | 0.080315 | 1197.3 | 917.43 | 2306.1 | 2.84 |
| Alistipes | 0.03163 | 0.084346 | 59823 | 73666 | 40312 | 4.22 |
| Sellimonas | 0.034132 | 0.08787 | 3219.9 | 8184.7 | 1213.8 | 3.54 |
| Ruminococcaceae | 0.035148 | 0.08787 | 256560 | 269540 | 193180 | 4.58 |
| Odoribacter | 0.039438 | 0.095606 | 2255 | 4466.5 | 3704.7 | 3.04 |
| Bradyrhizobium | 0.043033 | 0.10126 | 394.38 | 437.67 | 793.68 | 2.3 |
| Allisonella | 0.058707 | 0.13419 | 1731.2 | 2190.8 | 1027 | 2.77 |
| Sarcina | 0.061071 | 0.13426 | 2889.5 | 2473.6 | 1796.6 | 2.74 |
| Tyzzerella | 0.062097 | 0.13426 | 14373 | 17308 | 12995 | 3.33 |
| Parasutterella | 0.067974 | 0.1431 | 45303 | 55813 | 27142 | 4.16 |
| Megasphaera | 0.091845 | 0.1884 | 47106 | 8493.4 | 30002 | 4.29 |
| Butyricicoccus | 0.10167 | 0.20137 | 17907 | 23675 | 21299 | 3.46 |
| Bacteroides | 0.1032 | 0.20137 | 1750700 | 2191600 | 2537000 | 5.59 |
| Faecalibacterium | 0.10582 | 0.20155 | 1172600 | 1220400 | 837790 | 5.28 |
| Megamonas | 0.11617 | 0.21614 | 125230 | 117840 | 289880 | 4.93 |
| Eisenbergiella | 0.12045 | 0.219 | 2972.5 | 2282.2 | 879.8 | 3.02 |
| Not_Assigned | 0.13531 | 0.24055 | 1388600 | 1438000 | 1179700 | 5.11 |
| Sutterella | 0.14037 | 0.24413 | 10521 | 10991 | 7910.9 | 3.19 |
| Veillonella | 0.14474 | 0.24451 | 122000 | 49797 | 237060 | 4.97 |
| Dialister | 0.14746 | 0.24451 | 72255 | 100970 | 122270 | 4.4 |
| Desulfovibrio | 0.14976 | 0.24451 | 11596 | 19405 | 5369.3 | 3.85 |
| Catenibacterium | 0.16984 | 0.27175 | 5936.9 | 21177 | 851.45 | 4.01 |
| Oscillibacter | 0.18041 | 0.283 | 5238.8 | 7833.6 | 5381 | 3.11 |
| Eggerthella | 0.19438 | 0.29904 | 15230 | 2156.8 | 2620.6 | 3.82 |
| Holdemanella | 0.23258 | 0.34049 | 57574 | 29296 | 25421 | 4.21 |
| Roseburia | 0.23642 | 0.34049 | 208780 | 179060 | 159250 | 4.39 |
| Fusobacterium | 0.23901 | 0.34049 | 68467 | 6710.4 | 16717 | 4.49 |
| Butyricimonas | 0.24032 | 0.34049 | 1503.5 | 1771.9 | 2817 | 2.82 |
| Alloprevotella | 0.2426 | 0.34049 | 78737 | 39349 | 29056 | 4.4 |
| Dorea | 0.27712 | 0.38224 | 49716 | 46275 | 37235 | 3.8 |
| Lactobacillus | 0.28191 | 0.38225 | 67222 | 126920 | 112200 | 4.47 |
| Weissella | 0.28997 | 0.38663 | 4704.8 | 3512 | 3977 | 2.78 |
| Hungatella | 0.33116 | 0.43431 | 8198.3 | 12538 | 16615 | 3.62 |
| Parabacteroides | 0.40687 | 0.52499 | 122250 | 193830 | 180320 | 4.55 |
| Acidaminococcus | 0.4483 | 0.56927 | 3148.5 | 2176 | 2553.4 | 2.69 |
| Streptococcus | 0.47286 | 0.58923 | 254660 | 134680 | 350640 | 5.03 |
| Terrisporobacter | 0.48604 | 0.58923 | 17200 | 21542 | 15522 | 3.48 |
| Agathobacter | 0.48748 | 0.58923 | 3341.9 | 5118.1 | 2961.3 | 3.03 |
| Anaerostipes | 0.49949 | 0.58923 | 58759 | 37210 | 49820 | 4.03 |
| Mitsuokella | 0.50085 | 0.58923 | 5197.6 | 4644.7 | 6393.5 | 2.94 |
| Barnesiella | 0.51492 | 0.597 | 12893 | 12171 | 13621 | 2.86 |
| Coprobacillus | 0.5456 | 0.62354 | 10308 | 1619 | 1751 | 3.64 |
| Bifidobacterium | 0.57918 | 0.6526 | 137530 | 105970 | 75411 | 4.49 |
| Erysipelotrichaceae | 0.59571 | 0.66191 | 44830 | 38321 | 28581 | 3.91 |
| Subdoligranulum | 0.62775 | 0.68728 | 114660 | 102350 | 105790 | 3.79 |
| Actinomyces | 0.63573 | 0.68728 | 1897.9 | 1605.6 | 1824 | 2.17 |
| unidentified_Lachnospiraceae | 0.67326 | 0.71815 | 337500 | 357560 | 408840 | 4.55 |
| Collinsella | 0.68747 | 0.72365 | 27891 | 16484 | 70927 | 4.43 |
| Flavonifractor | 0.72279 | 0.75095 | 17257 | 15173 | 18854 | 3.27 |
| Senegalimassilia | 0.80389 | 0.8245 | 1270.7 | 1786.5 | 804.34 | 2.69 |
| Enterobacteriaceae | 0.94911 | 0.9508 | 652200 | 420920 | 692160 | 5.13 |
| Lachnoclostridium | 0.9508 | 0.9508 | 144870 | 129690 | 128440 | 3.91 |

FDR, False Discovery Rate; HCC, Hepatocellular carcinoma; LDA, linear discriminant analysis.

**Table S4. Different degree of genera levels in HCC with HBV, HCC with HCV and HCC with alcohol**

| **Feature** | **P values** | **FDR** | **HCC with HCV** | **HCC with alcohol** | **HCC with HBV** | **LDA score** |
| --- | --- | --- | --- | --- | --- | --- |
| Enterococcus | 1.06E-07 | 4.64E-06 | 8655.6 | 45442 | 65991 | 4.46 |
| Papillibacter | 1.18E-07 | 4.64E-06 | 1571 | 1323.2 | 208.06 | 2.83 |
| Faecalitalea | 1.62E-07 | 4.64E-06 | 576.37 | 10526 | 246.79 | 3.71 |
| Megasphaera | 4.96E-07 | 1.07E-05 | 6690.3 | 3802.9 | 69275 | 4.52 |
| Phyllobacterium | 2.08E-06 | 3.58E-05 | 25327 | 923.5 | 28612 | 4.14 |
| Sutterella | 5.74E-06 | 7.75E-05 | 13162 | 14427 | 7198.1 | 3.56 |
| Catenibacterium | 6.63E-06 | 7.75E-05 | 20041 | 11782 | 3199.7 | 3.93 |
| Coprobacillus | 7.21E-06 | 7.75E-05 | 5596.2 | 28112 | 255.16 | 4.14 |
| Negativibacillus | 1.41E-05 | 0.000127 | 738.35 | 864.61 | 141.82 | 2.56 |
| Mitsuokella | 1.48E-05 | 0.000127 | 2756 | 3810.5 | 7174.6 | 3.34 |
| Ruminococcaceae | 2.77E-05 | 0.000217 | 22136 | 29207 | 7565.2 | 4.03 |
| Lachnospiraceae | 3.51E-05 | 0.000252 | 3486.7 | 411.02 | 3805 | 3.23 |
| Planktomarina | 0.000201 | 0.001017 | 21072 | 22.515 | 379.08 | 4.02 |
| Rothia | 0.001521 | 0.004887 | 420.82 | 1162.9 | 1159.2 | 2.57 |
| Paraprevotella | 0.007617 | 0.017705 | 32532 | 12861 | 78795 | 4.52 |
| Roseburia | 0.0146 | 0.029199 | 232260 | 133230 | 203500 | 4.69 |
| Akkermansia | 0.017983 | 0.034368 | 31772 | 5662.8 | 15591 | 4.12 |
| Bradyrhizobium | 0.023579 | 0.043144 | 603.99 | 101.05 | 393.15 | 2.4 |
| Intestinimonas | 0.033357 | 0.059764 | 12060 | 9321.2 | 7033.4 | 3.4 |
| Sarcina | 0.054063 | 0.091165 | 2265.6 | 985.79 | 3881 | 3.16 |
| Eisenbergiella | 0.070713 | 0.11695 | 1707.7 | 3135.7 | 3400.5 | 2.93 |
| Peptostreptococcus | 0.09086 | 0.14743 | 309.6 | 721.85 | 274.35 | 2.35 |
| Gemella | 0.098918 | 0.15754 | 710.62 | 1464.9 | 1238.7 | 2.58 |
| Delftia | 0.10667 | 0.16381 | 4698.8 | 3363 | 3346 | 2.83 |
| Dialister | 0.10949 | 0.16495 | 104920 | 66554 | 70341 | 4.28 |
| Dorea | 0.11125 | 0.16495 | 48926 | 52515 | 46504 | 3.48 |
| Terrisporobacter | 0.13767 | 0.20067 | 22363 | 24368 | 13131 | 3.75 |
| Turicibacter | 0.15681 | 0.22476 | 269550 | 325780 | 229580 | 4.68 |
| Butyricimonas | 0.18635 | 0.26272 | 2041.4 | 1065 | 1458.1 | 2.69 |
| Allisonella | 0.22611 | 0.30866 | 1546 | 1531.3 | 2251.5 | 2.56 |
| Fusicatenibacter | 0.23998 | 0.32248 | 72564 | 136240 | 86009 | 4.5 |
| Lactococcus | 0.24686 | 0.32662 | 11053 | 211.21 | 818.93 | 3.73 |
| Bilophila | 0.27185 | 0.34994 | 12520 | 8428.7 | 7501.2 | 3.4 |
| Blautia | 0.27263 | 0.34994 | 333480 | 511330 | 592150 | 5.11 |
| Tyzzerella | 0.31792 | 0.40207 | 18143 | 12268 | 14371 | 3.47 |
| Bacteroides | 0.45278 | 0.54844 | 1694700 | 1692800 | 2095800 | 5.3 |
| Hungatella | 0.46356 | 0.5537 | 6212.7 | 8354.8 | 12365 | 3.49 |
| Subdoligranulum | 0.48195 | 0.56778 | 110290 | 79565 | 124010 | 4.35 |
| Lachnoclostridium | 0.50069 | 0.58188 | 168400 | 140600 | 118460 | 4.4 |
| Holdemanella | 0.51978 | 0.59602 | 63557 | 26714 | 47733 | 4.27 |
| Adlercreutzia | 0.57928 | 0.64699 | 3518.9 | 2238.1 | 4210.4 | 2.99 |
| Parasutterella | 0.57928 | 0.64699 | 23390 | 45096 | 69165 | 4.36 |
| Streptococcus | 0.76218 | 0.78972 | 262220 | 162650 | 208230 | 4.7 |
| Phascolarctobacterium | 0.77303 | 0.79144 | 170640 | 205250 | 174840 | 4.24 |
| Faecalibacterium | 0.82269 | 0.83237 | 1129900 | 1170400 | 1228800 | 4.69 |
| Prevotellaceae | 0.99543 | 0.99543 | 1148200 | 1405300 | 1024800 | 5.28 |

FDR, False Discovery Rate; HCC, Hepatocellular carcinoma; HCV, hepatitis C virus; HBV, hepatitis B virus; LDA, linear discriminant analysis.
